# PHR1 mediates rapid high light responses and acclimation to high photosynthetic activity

**DOI:** 10.1111/tpj.70901

**Published:** 2026-05-05

**Authors:** Lukas Ackermann, Agnes Fekete, Laura Schröder, Thomas Nägele, Alina J. Hieber, Monika Müller, Martin J. Mueller, Maria Klecker

**Affiliations:** ^1^ Plant Physiology University of Bayreuth 95447 Bayreuth Germany; ^2^ Julius‐von‐Sachs‐Institute of Biosciences, Biocenter, Pharmaceutical Biology University of Würzburg 97082 Würzburg Germany; ^3^ Faculty of Biology, Plant Evolutionary Cell Biology LMU München 82152 Planegg Germany

**Keywords:** PHR1, phosphate starvation, light acclimation, photosynthesis, SRG3/GDPD1, linolenic acid, photoprotection, triose phosphate utilization

## Abstract

Changing light intensity requires immediate metabolic adjustment which involves reprogramming of both plastidial and nuclear gene expression, but the signaling pathways behind such responses are not fully understood. Here we report that an increase in light intensity causes fluctuations of P_i_ levels in the chloroplasts of *Arabidopsis thaliana* and induces a transcriptional response mediated by the nuclear low‐P_i_ signaling machinery involving the transcription factor PHOSPHATE STARVATION RESPONSE 1 (PHR1). Activation of PHR1 was linked to triose phosphate metabolism since the double mutant *adg1 tpt‐2*, defective in both starch accumulation and triose phosphate export from the chloroplast, exhibited a constitutive transcriptional P_i_ starvation signature. Among the high light‐induced target genes of PHR1, we further investigated *SRG3*/*GDPD1*, encoding an enzyme involved in phospholipid catabolism. Lipid profiling revealed pronounced changes in *srg3* mutants compared with wild‐type plants, including high light triggered accumulation of α‐linolenic acid and reduced levels of the photoprotective carotenoid zeaxanthin. We propose that photosynthetic activity regulates the low‐P_i_ response machinery in the nucleus in order to implement high light acclimation leading to the liberation of cellular P as well as the maintenance of membrane integrity.

## Significance Statement

Photosynthesis transiently consumes inorganic phosphate (P_i_). This study demonstrates that increasing light intensity considerably affects chloroplast P_i_ levels and rapidly activates a nuclear transcriptional response mediated by the transcription factor PHR1 involved in low‐P_i_ signaling. Among the high light‐induced targets of PHR1, we ascribe a downstream role to *SRG3/GDPD1*, encoding a glycerophosphodiester phosphodiesterase, in the maintenance of membrane integrity and adequate accumulation of the xanthophyll zeaxanthin.

## INTRODUCTION

Light energy is provided to photoautotrophic organisms at variable intensities that can fall below or exceed the requirements of the photosynthetic machinery at unpredictable times during the day period. This necessitates an enzymatic machinery capable of coping with rapid changes in metabolic flux, as well as the containment of overexcitation‐induced hazards, such as lipid peroxidation (Havaux & Niyogi, 1999). Both challenges are met not only by post‐transcriptional regulation of enzyme activity (reviewed in König et al. (2012), Matiolli et al. (2022)), but also by pronounced changes in mRNA abundance that occur within 30 min upon an increase in irradiance (Huang et al., 2019; Suzuki et al., 2015; Vogel et al., 2014). The latter mechanism depends on fast signal transmission between the chloroplast and the nuclear transcription machinery. Such communication is mediated by ‘primary’ retrograde signals (Dietz, 2015), including redox cues and reactive oxygen species which are produced during photosynthetic reactions (Bechtold et al., 2008; Dietz et al., 2016). Additionally, increased electron transport activity and light‐induced Calvin–Benson cycle activation promote the accumulation of primary products of carbon fixation (Borghi et al., 2019; Dietz & Heber, 1984, 1986) which may themselves act as signaling molecules (Häusler et al., 2014; Moore et al., 2014). Hence, induction of high light‐specific gene expression partially depends on the triose phosphate/phosphate translocator (TPT) that mediates the exchange of triose phosphates and 3‐phosphoglycerate (3‐PGA) with inorganic phosphate (P_i_) at the chloroplast envelope (Schneider et al., 2002; Vogel et al., 2014; Weise et al., 2019). A central function of the TPT in light acclimation was corroborated by the severe high light‐dependent phenotypes of double mutants defective in *TPT* and *ADG1* (for *ADP GLUCOSE PYROPHOSPHORYLASE 1*), encoding a small subunit of the ADP–glucose pyrophosphorylase complex (AGPase) which is required for the biosynthesis of transitory starch (Schmitz et al., 2012). While photosynthate partitioning plays a crucial role in retrograde signaling, the identity of the primary sensors as well as signal transduction mechanisms are still unclear.

Phosphorus makes up about 2–30 permille of the plant dry mass (Kumar et al., 2019), as it is incorporated into a large subset of biomolecules. Systemic signaling of P_i_ availability and P_i_ distribution within the plant body mainly depend on a conserved gene family encoding PHOSPHATE STARVATION RESPONSE 1 (PHR1) and at least 14 PHR1 LIKE (PHL) transcription factors of the GARP coiled‐coiled type (Bustos et al., 2010; Rubio et al., 2001; Safi et al., 2017; Sun et al., 2016; Thibaud et al., 2010; Zhou et al., 2008). Simultaneous loss of *PHR1* and *PHL1* function affects roughly 70% of the transcriptional P_i_ starvation response (PSR) in the shoots of Arabidopsis (*Arabidopsis thaliana*, L.) (Bustos et al., 2010). A decrease in cellular P_i_ concentration impairs production of the inositol pyrophosphate species InsP8 in the cytosol, relieving PHR1/PHLs of suppression by SPX (for SYG1/Pho81/XPR1) domain‐containing proteins, thus linking transcription factor activity to P_i_ availability (Dong et al., 2019; Puga et al., 2014; Ried et al., 2021; Zhu et al., 2019). The transcriptional response to P_i_ scarcity leads to scavenging of external and internal P_i_ sources through phosphatase induction (Morcuende et al., 2007) and altered lipid metabolism (Misson et al., 2005; Pant et al., 2015; Yang et al., 2025), anthocyanin biosynthesis (Li et al., 2023; Liu et al., 2022; Nilsson et al., 2012), and protection from photodamage (Nilsson et al., 2012), to name a selection. The largest pool of cellular P reserves resides in the vacuole, and it has been shown in rice (*Oryza sativa*, L.) that vacuolar P_i_ efflux into the cytosol is mediated by two proteins of the glycerol 3‐phosphate transporter family which are transcriptionally induced by PHR proteins (Xu et al., 2019). Three homologs have been identified in Arabidopsis and are termed VPE1‐3 (Ramaiah et al., 2011; Xu et al., 2019), but their contribution to P_i_ homeostasis has not been addressed so far. Direct targets of PHR1 are strongly upregulated upon P_i_ starvation and include genes such as *PHOSPHATE STARVATION‐INDUCED GENE 2* (*PS2*; AT1G73010) (Hanchi et al., 2018), *MONOGALACTOSYLDIACYLGLYCEROL SYNTHASE 3* (*MGD3*; AT2G11810) (Kobayashi et al., 2004), and *GLYCEROPHOSPHODIESTER PHOSPHODIESTERASE 1/SENESCENCE‐RELATED GENE 3* (*GDPD1*/*SRG3*; AT3G02040) (Cheng et al., 2011). The latter encodes a plastid‐localized enzyme with glycerophosphodiester phosphodiesterase activity acting on a broad spectrum of deacylated phospholipids (Cheng et al., 2011). SRG3 is supposed to mainly function in the catabolism of phosphatidylcholine to provide glycerol‐3‐phosphate for DGDG biogenesis, for glycolytic utilization, or liberation of P_i_ by subsequent acid phosphatase activity (Cheng et al., 2011; Wojciechowska et al., 2024). The *SRG3* gene was shown to be important for seedling fitness and maintenance of cellular P_i_ levels under P_i_ starvation conditions (Cheng et al., 2011), but how exactly SRG3 contributes to nutrient homeostasis is not understood.

P_i_ is unique among the macronutrients in that it is the only root‐supplied element which is directly required for photosynthesis (Dietz & Heber, 1984; Heldt & Rapley, 1970; Sivak & Walker, 1986). In this study, we show that increments in light intensity rapidly disturb subcellular P_i_ levels *in planta*. Strikingly, this is accompanied by a nuclear transcriptional response characteristic of P_i_ depletion which depends on *PHR1*. Transcript analysis of the *adg1 tpt‐2* mutant links PHR1 activation to triose phosphate allocation. Low‐P_i_ mediated signaling was relevant for light acclimation since *phr1‐1 phl1* mutants were impaired in high light‐mediated anthocyanin production. Moreover, induction of genes involved in lipid metabolism such as *SRG3* by PHR1 contributes to membrane photoprotection and to regulation of the levels of free linolenic acid, as revealed by lipid profiling of the *srg3* mutant upon high light. Together, we propose that PHR1 is activated during triose phosphate overproduction, and mediates acclimation to higher light intensity through cellular P_i_ release and changes in lipid metabolism.

## RESULTS

### Triose phosphate utilization affects P_i_ signaling

**Figure 1:**
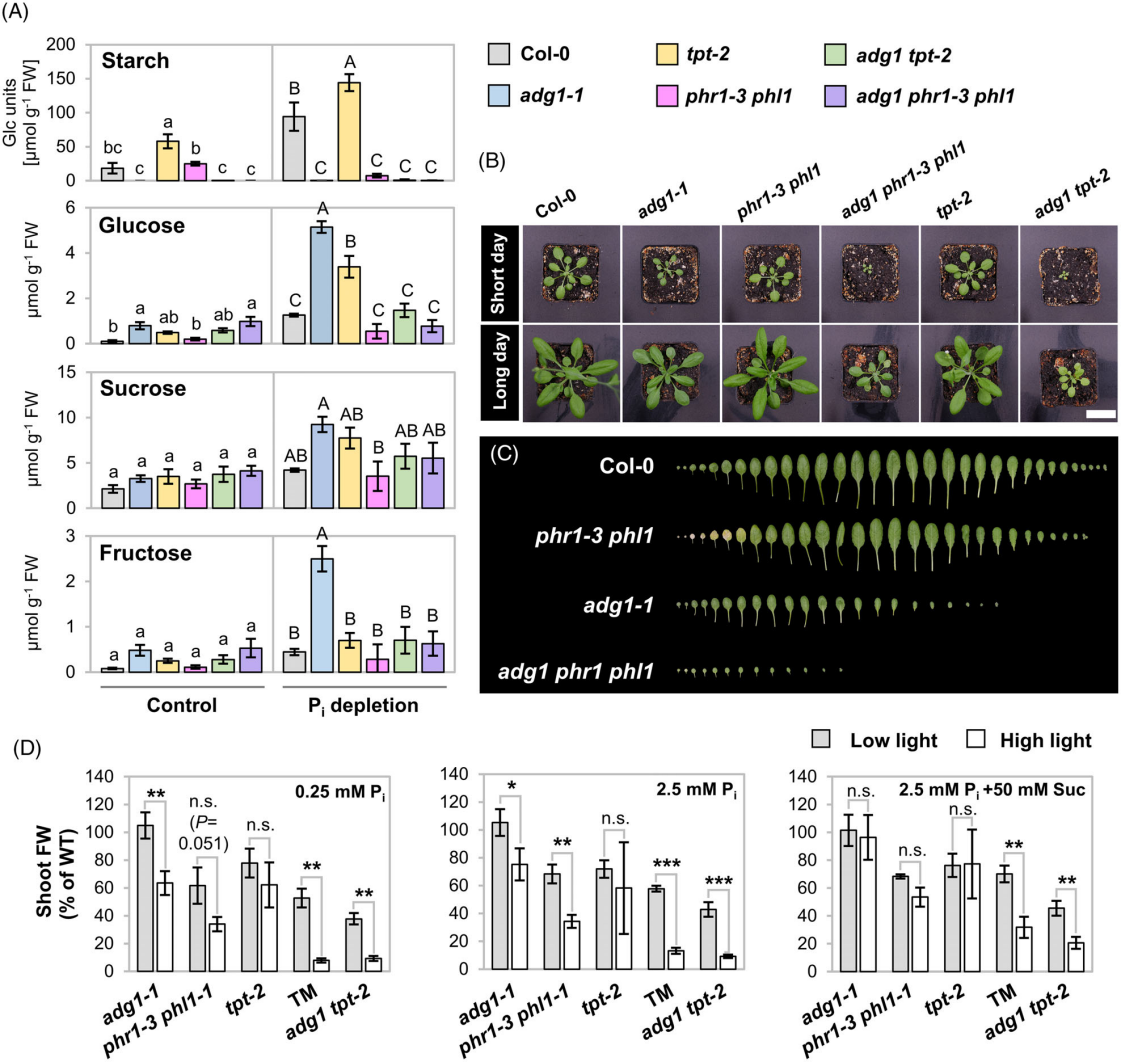
Mutants of photoassimilate partitioning and P_i_ signaling exhibit synergistic phenotypes. (A) Contents of carbohydrates in shoots upon P_i_ depletion relative to fresh weight (FW). Seedlings of wild‐type (WT) (Col‐0) and indicated mutant genotypes were grown for 10 days on rich medium before transfer to media with either 2.5 mM (Control) or 0 mM (P_i_ depletion) KH_2_PO_4_ added. Growth was continued for 7 days. Pools of seedling shoots were harvested 10.25 h after onset of the photoperiod. *n* = 3 independent experiments. One‐way anova with Tukey HSD follow‐up test and Bonferroni alpha correction for contrasts; statistical analysis was performed individually for each growth condition, indicated by different letter case. Different letters indicate statistical differences in genotypes (*P* < 0.05). (B, C) Phenotype of the *adg1 phr1 phl1* triple mutant. Plants were grown at a light intensity of 90 ± 10 μmol m^−2^ sec^−1^. (B) Rosette habitus of plants grown under short‐day (8 h light period; 27‐days age) and long‐day (16 h light period; 25 days age) conditions. Bar, 2.5 cm. (C) Leaves of 42‐day‐old plants grown under short day were aligned according to age (starting with the cotyledons on the left hand side). Background was manually removed for better visualization. (D) High light sensitivity of *phr1‐3 phl1* and *adg1 phr1 phl1* (TM) seedlings can be alleviated by exogenous sucrose. Seedlings were grown for 2 weeks under low light (60 ± 5 μmol m^−2^ sec^−1^) or high light (350 ± 30 μmol m^−2^ sec^−1^) on media containing 0.25 mM P_i_, 2.5 mM P_i_, or 2.5 mM P_i_ + 50 mM sucrose. Shoot FW were calculated as percent of mean shoot FW of WT seedlings. *n* = 3 independent experiments comprising means of each 4–8 seedling shoots. Student's *t*‐test with two‐tailed distribution, unpaired with unequal variance; ***P < 0.001, **P < 0.01, *P < 0.05, n.s., not significant. All bars are means ± standard deviations.

Photoassimilate allocation and utilization in the chloroplast and cytosol are supposed to limit the rate of P_i_ recycling from photosynthetic products (McClain & Sharkey, 2019). In line with this assumption, P_i_ deprivation has been shown to stimulate starch accumulation in a *PHR1*‐dependent manner, which involves AGPase activity (Nilsson et al., 2007). To address whether partitioning of phosphorylated assimilates is important for growth during P_i_‐limiting conditions, we examined the phenotypes of mutants defective in starch biosynthesis (*adg1‐1*) and chloroplastic triose phosphate export (*tpt‐2*) during P_i_ starvation in the presence of sucrose. Starch accumulation in response to P_i_ depletion was abolished in both the *phr1‐3 phl1* double mutant and the *adg1‐1* mutant (Figure 1A). Concomitantly, P_i_‐depleted *adg1‐1* mutants accumulated high levels of monosaccharides (Figure 1A). Loss of TPT function caused significantly higher levels of starch and glucose under P_i_ deprivation as compared with the wild‐type (WT), which resumed a trend that is also observed under control conditions (Schneider et al., 2002). Interestingly, overaccumulation of carbohydrates under P_i_ deprivation was ceased in the double mutant *adg1 tpt‐2*. Likewise, *adg1 tpt‐2* double mutants failed to accumulate anthocyanins upon P_i_ deprivation (Figures S1A,B).

In order to test if nuclear gene regulation by PHR1/PHL1 is important for plant growth when P_i_ recycling from photosynthetic intermediates is impaired, we generated the triple mutant *adg1‐1 phr1‐3 phl1* (hereafter referred to as *adg1 phr1 phl1*). This mutant exhibited a pronounced growth defect which was the result of reduced leaf size and number (Figure 1B,C). Next, *adg1 phr1 phl1* plants flowered later than the parental lines (Figure S1C). These findings indicate that transcriptional regulation by PHR1/PHL1 aids growth when starch production is prevented.

We noted that growth retardation of *adg1 phr1 phl1* resembled the phenotype of the *adg1 tpt‐2* mutant (Figure 1B). It was reported that the *adg1 tpt‐2* mutant is sensitive toward higher light intensities, which could be overcome by exogenously supplied sucrose (Heinrichs et al., 2012; Schmitz et al., 2012). We therefore asked if the growth defect of *adg1 phr1 phl1* was also dependent on light intensity. In fact, high light‐dependent growth restriction of *adg1 phr1 phl1* seedlings was comparable to *adg1 tpt‐2*, both under low and high P_i_ conditions (Figure 1D; Figure S2). As for *adg1 tpt‐2*, the difference in shoot (Figure 1D) and root (Figure S2) growth performance between low‐ and high light conditions was alleviated by providing sucrose to *adg1 phr1 phl1* mutants. Interestingly, a less pronounced high light‐dependent growth restriction was also seen for both the *adg1‐1* and *phr1‐3 phl1* mutants (Figure 1D). Again, this was attenuated by exogenous sucrose.

**Figure 2:**
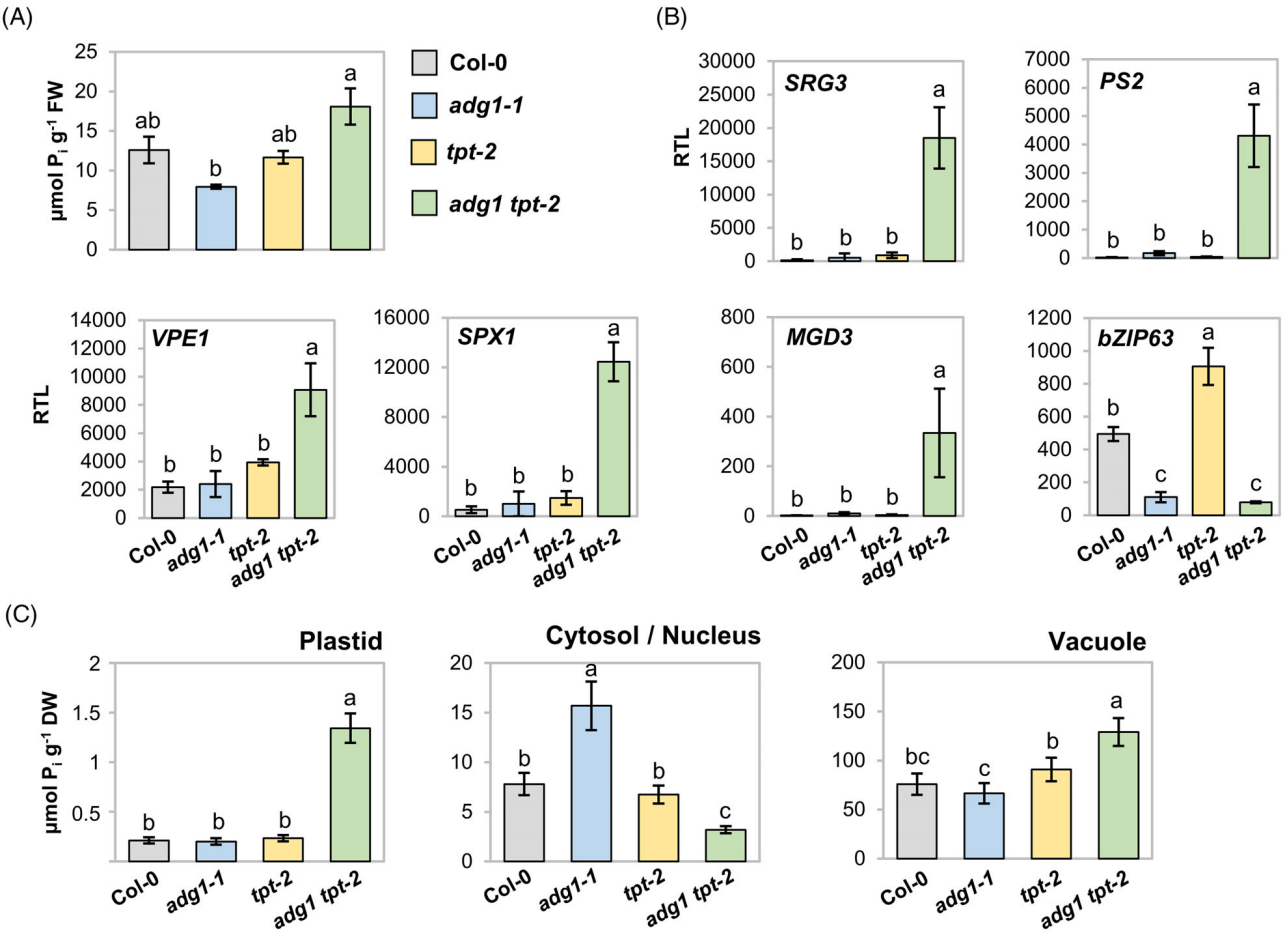
The *adg1 tpt‐2* mutant shows altered P_i_ distribution and signaling. Plants were grown on soil and sampled 135 min after onset of the 8‐h photoperiod after 5 weeks of growth. All bars are means ± standard deviations. (A) Total P_i_ contents of rosette leaves. *n* = 3 independent experiments. Kruskal–Wallis test with DUNN's follow‐up test and DUNN/Sidak alpha correction for contrasts; different letters indicate statistical differences in genotypes (*P* < 0.01). (B) qRT‐PCR analysis of P_i_ starvation‐responsive genes in rosette leaves of wild‐type (WT) (Col‐0), *adg1‐1*, *tpt‐2*, and *adg1 tpt‐2*. Relative transcript levels (RTL) are 1000 × 2^−ΔCT^ with *PP2A* as the reference gene. *n* = 3 independent experiments. (C) Subcellular P_i_ contents in rosettes were determined relative to plant dry weight after non‐aqueous fractionation. *n* = 3–5 pools of rosettes from three independent trials. (B, C) One‐way anova with Tukey HSD follow‐up test and Bonferroni alpha correction for contrasts; different letters indicate statistical differences in genotypes at *P* < 0.01 (B) and *P* < 0.05 (C).

Our data indicated that carbohydrate allocation and P_i_ signaling are interconnected. To further test if the mutation of *adg1 tpt‐2* affects cellular P_i_ signaling, we measured P_i_ contents of aboveground tissues and determined P_i_ starvation gene expression in vegetative plants grown on soil. Surprisingly, *adg1 tpt‐2* double mutants overaccumulated P_i_ compared with the *adg1‐1* single mutant (Figure 2A). Counter‐intuitively, quantitative reverse transcriptase‐polymerase (qRT‐PCR) analysis revealed that transcripts of PSR marker genes, including *SRG3*, *PS2*, *VPE1* (AT3G47420), *SPX1*, and *MGD3* (Figure S3), were all constitutively upregulated in *adg1 tpt‐2* (Figure 2B). Additionally, the transcript abundance of the circadian regulator *BASIC LEUCINE ZIPPER 63* (*bZIP63*) (Baena‐González et al., 2007; Frank et al., 2018), which is repressed by P_i_ depletion in the WT (Figure S3), was also reduced in rosettes of *adg1 tpt‐2* as well as *adg1‐1* (Figure 2B). P_i_ signaling relates to the P_i_‐dependent metabolism of InsPs in the cytosol (Wild et al., 2016). Since *adg1 tpt‐2* mutants are defective in major pathways of P_i_ recycling executed in the chloroplast and cytosol, we asked whether disturbed P_i_ signaling and accumulation are caused by altered subcellular distribution of P_i_ in this mutant. To address this, we determined P_i_ levels after performing non‐aqueous fractionations of plant rosettes. Strikingly, *adg1 tpt‐2* plants contained increased plastidial pools of P_i_ compared with the control lines, while P_i_ levels in the cytosolic/nuclear fractions were reduced, which would be consistent with InsP‐dependent PHR1 activation (Figure 2C). Vacuolar stores of P_i_ in *adg1 tpt‐2* were also higher than in any of the control lines, accounting for the bulk of the increased total P_i_ pools in the mutant.

We concluded from these experiments that triose phosphate allocation has pronounced effects on P_i_ distribution, including the regulation of cytosolic/nuclear P_i_ concentrations, and on the regulation of the nuclear PSR. Moreover, PHR1/PHL1 signaling is important for normal plant growth when triose phosphate availability is high.

### High light elicits PSR gene expression

**Figure 3:**
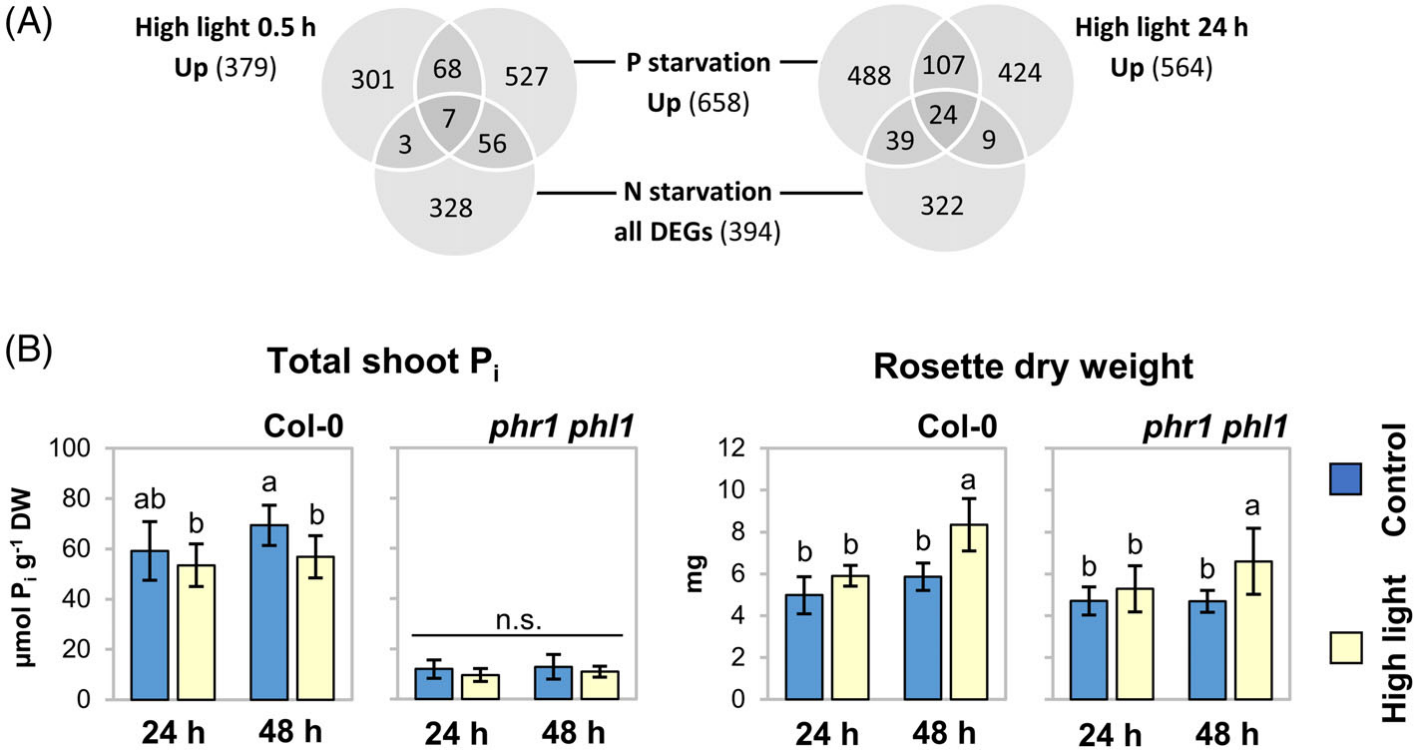
High light triggers response to P_i_‐depletion. (A) Overlap of differential gene expression in shoots upon high light, P_i_ starvation, and nitrogen starvation. Venn diagrams show numbers of transcripts upregulated by high light or P_i_ starvation, and of genes differentially expressed under N starvation. Transcript data are taken from Huang et al. (2019) (high light), Bustos et al. (2010) (P_i_ depletion), and Krapp et al. (2011) (N depletion). (B) High light affects total shoot P_i_ and growth rates on the long term. Wild‐type (WT) (Col‐0) and *phr1‐1 phl1* mutants were grown on soil for 5 weeks before light intensity was shifted to 450 ± 30 μmol m^−2^ sec^−1^ (high light, yellow bars) at 4 h after onset of the photoperiod. Control plants were kept under growth light conditions (70 ± 5 μmol m^−2^ sec^−1^; blue bars). Material was harvested after 24 and 48 h. Total P_i_ contents in rosettes relative to DW and average rosette DWs are depicted; *n* = 10 plants from five individual experiments. Bars depict means ± standard deviations; letter code indicates statistical differences at *P* < 0.05 based on two‐factor anova with Tukey's HSD *post‐hoc* test.

The results of our reverse genetic analyses indicated that PSR gene expression is affected by photoassimilate utilization. This raised the question of whether P_i_ signaling was also modulated in WT plants under non‐starved conditions by naturally occurring changes in photoassimilate availability such as upon a shift in light intensity. In order to address this, we interrogated published transcriptomic data collected from seedling shoots under P_i_ starvation (Bustos et al., 2010) and high light treatments under nutrient‐rich conditions (Huang et al., 2019). We found that out of 379 transcripts upregulated (log_2_ fold change >2) by 30 min of high light, 75 were also upregulated by P_i_ starvation (Figure 3A). For longer high light treatments of 24 h, 131 out of 564 transcripts were also P_i_‐responsive (Figure 3A). In both cases, the overlap was found to be significant at *P* < 0.0001 in hypergeometric testing using Fisher's exact test. In order to estimate whether the observed overlap might be due to generally higher nutrient requirements under high light conditions, we next included a set of nitrogen‐responsive transcripts in the comparison (Krapp et al., 2011). Crucially, nitrogen‐responsive transcripts were not significantly enriched among high light‐induced transcripts at 30 min high light (10 out of 379), but that was the case at 24 h (33 out of 564; Figure 3A). Hence, to address if PSR gene expression upon longer duration of high light exposure could be attributable to enhanced nutrient requirements, we determined total P_i_ contents of rosettes subjected to 24 and 48 h of light increase. In fact, shoot P_i_ levels of WT plants were slightly reduced within 48 h of high light as compared with control light conditions (Figure 3B). This correlated with significantly enhanced plant biomass measured upon 48 h of high light compared with control treatments (Figure 3B), suggesting that light increase stimulated growth which was not immediately satisfied by additional nutrient acquisition. In contrast to WT plants, P_i_ contents in leaves of *phr1‐1 phl1* mutant plants remained at low levels independently of the light treatment (Figure 3B). Thus, high light stress seems to elevate nutrient demand of the plant in the long term, but low‐P_i_ transcription is specifically triggered already at an early time point of treatment.

### High light‐induced PSR gene expression depends on *PHR1*

**Figure 4:**
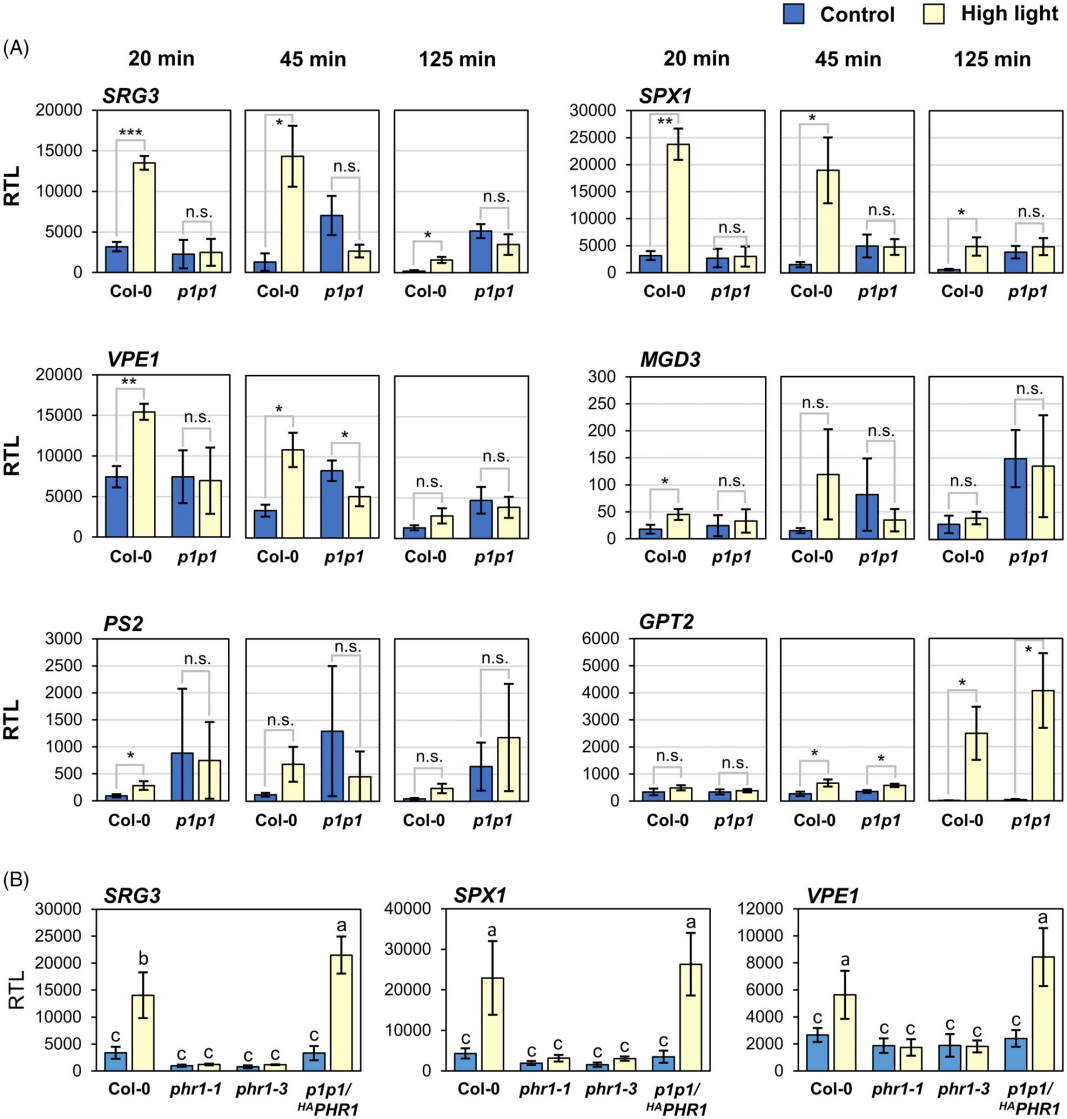
High light‐induced PSR gene expression depends on *PHR1*. Plants were grown on fertilized soil in an 8 h/16 h (light/dark) photoregime and exposed to high light (450 ± 30 μmol m^−2^ sec^−1^, yellow bars) starting 1 h after onset of the photoperiod at 36‐days age. The control group was kept at 70 ± 5 μmol m^−2^ sec^−1^ (blue bars). 2–3 fully developed leaves (leaves no. 10–13) were harvested per sample. (A) qRT‐PCR analyses of selected transcripts in leaves from wild‐type (WT) (Col‐0) and *phr1‐1 phl1 (p1p1)* mutants at 20, 45, and 125 min of high light stress. *n* = 3 independent experiments. Statistical analyses were performed using Student's *t*‐test with two‐tailed distribution, unpaired with unequal variance. Significant differences are indicated as ***P < 0.001, **P < 0.01, *P < 0.05, n.s., not significant. (B) qRT‐PCR analysis of *SRG3, SPX1, and VPE1* transcripts in rosette leaves from WT (Col‐0), *phr1‐1* and *phr1‐3* single mutants, and the transgenic line *phr1‐1 ph11*/*HA‐PHR1* (#8 shown in Figure S5B). 30 min of high light treatment were applied; *n* = 3 independent experiments. Statistical analyses were performed using two‐factor anova with Tukey's HSD *post‐hoc* test; different letters indicate statistical differences (*P* < 0.05). All transcript levels were calculated relative to *PP2A* as 1000 × 2^−ΔCT^. All bars represent means ± standard deviations.

We asked whether the induction of PSR genes by short‐term high light required the activity of PHR1(‐like) transcription factors and thus was truly caused by low‐P_i_ signaling pathways. We grew WT and *phr1‐1 phl1* mutant plants under nutrient‐rich conditions on soil in an 8/16 h (light/dark) photoregime. After 5 weeks, the photon flux density was increased to 450 ± 30 μmol m^−2^ sec^−1^ (high light) at 1 h after commencement of the photoperiod, while the control group was kept under growth light conditions of 70 ± 5 μmol m^−2^ sec^−1^. We conducted qRT‐PCR analysis of samples harvested after 20, 45, and 125 min of treatment and detected pronounced upregulation of *SRG3*, *SPX1*, and *VPE1* transcripts in WT plants already after 20 min of exposure to high light, as well as a slight increase in *MGD3* and *PS2* transcript abundance (Figure 4A). In all five cases, gene expression was attenuated within 125 min in high light. Crucially, PSR marker transcripts were not responsive to light conditions in *phr1‐1 phl1* mutant plants (Figure 4A), suggesting that PHR1/PHL1 are required for high light‐mediated regulation of these genes. Notably, high light induction of *GLUCOSE‐6‐PHOSPHATE/PHOSPHATE TRANSLOCATOR 2* (*GPT2*), which is responsive to both light (Athanasiou et al., 2010) and P_i_ depletion (Morcuende et al., 2007), was not affected in *phr1‐1 phl1* mutants (Figure 4A), verifying that the response to high light is not generally impaired in the double mutant. In line with *PHR1*‐independent induction of *GPT2*, we were not able to detect an effect of the *gpt2‐1* allele (Niewiadomski et al., 2005) on carbohydrate accumulation under P_i_ depletion (Figure S4), arguing against a major role of this gene for sugar homeostasis under P_i_ starvation.

*PHR1* expression itself was reported to depend on light (Liu et al., 2017). Importantly, however, increasing the light intensity did not affect *PHR1* transcript levels under our experimental conditions (Figure S5A), consistent with the accumulation of *PHR1* transcript being only responsive to changes in the very low fluence range (Liu et al., 2017). Moreover, we created transgenic lines expressing HA‐tagged PHR1 under the control of a *PHR1* promoter fragment in *phr1‐1 phl1* double mutants. Protein levels of HA‐PHR1 were not changed by high light treatment in any of the three tested independent transgenic lines (Figure S5B), confirming that high photosynthetic radiation does not impact PHR1 abundance.

To further test the individual contribution of *PHR1* to the high light‐induced PSR, we also assessed gene expression in *phr1* single mutants (*phr1‐1* and *phr1‐3*), as well as in one of the *phr1‐1 phl1*/*HA‐PHR1* transgenic lines (line #8 in Figure S5B). In fact, transcript accumulation of *SRG3*, *SPX1*, and *VPE1* was abolished in both single mutants, and ectopic *HA‐PHR1* expression fully rescued gene induction in the double‐mutant background (Figure 4B), showing that *PHR1* was essential, while *PHL1* was dispensable for this response. In conclusion, high light treatment at early time points triggers low‐P_i_ gene expression mediated by post‐translational activation of PHR1, resulting in the transient upregulation of transcripts such as *SRG3*, *SPX1*, and *VPE1*.

### Short‐term high light affects chloroplast P_i_ levels and sucrose accumulation

**Figure 5:**
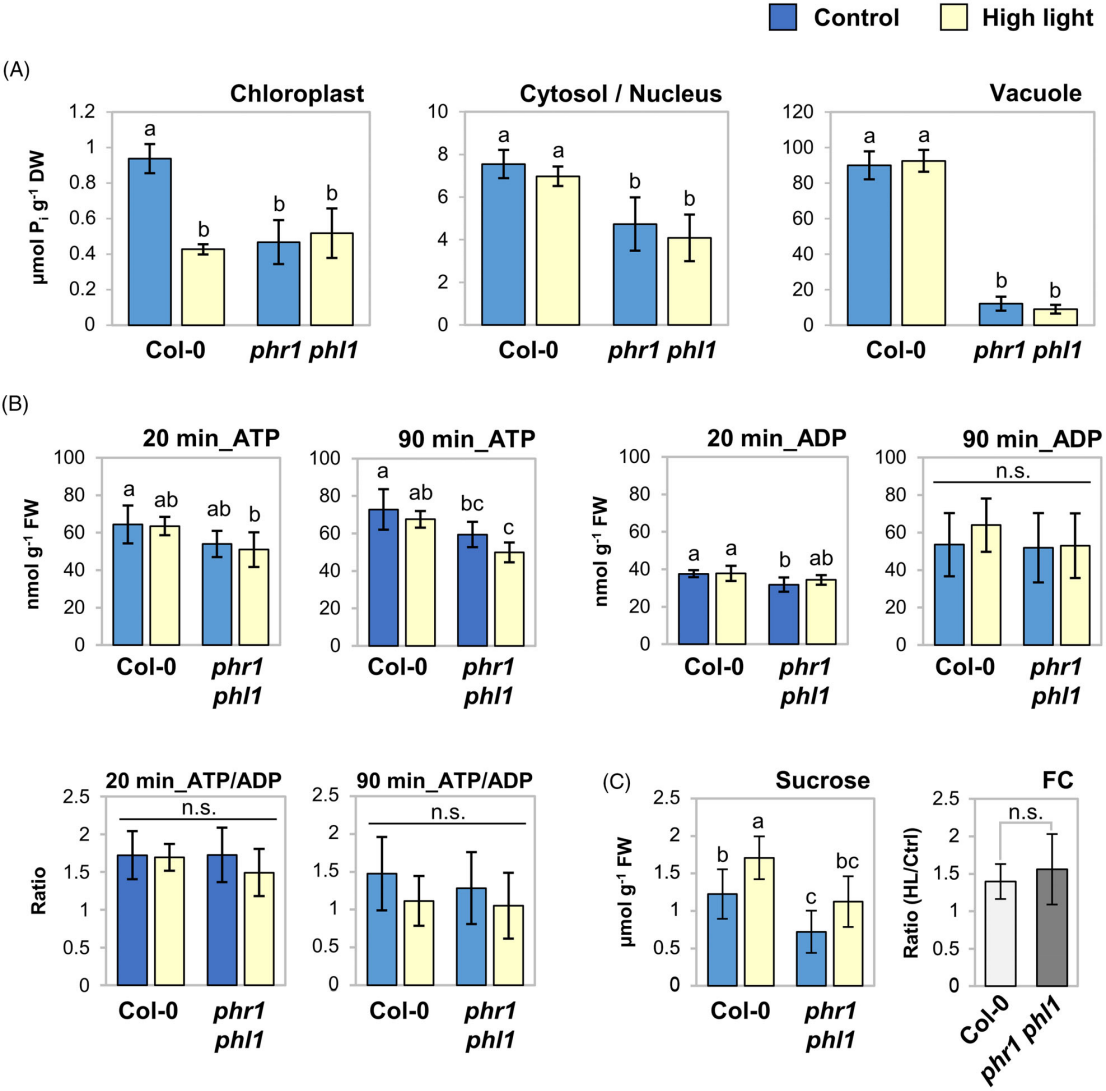
High light affects plastidial P_i_ pools and sucrose levels. Plants were grown and treated as described in Figure 4. (A) Subcellular P_i_ concentrations in wild‐type (WT) and *phr1‐1 phl1* mutant plants upon high light relative to dry weight (DW). Rosettes were harvested after 20 min of treatment. P_i_ contents were determined following non‐aqueous fractionation of cellular compartments. *n* = 4 (*phr1‐1 phl1*) or 5 (Col‐0) independent experiments. (B) Changes in ATP/ADP levels after shift to high light relative to fresh weight (FW). *n* = 6 plants from three (90 min) or four (20 min) independent experiments. (C) Sucrose levels upon 20 min of high light; *n* = 8 plants from four independent experiments; absolute values and fold changes (FC, high light/control) are shown. Significance in FC was tested using Student's *t*‐test with two‐tailed distribution, unpaired with unequal variance. All bars show means ± standard deviations. Except for FC shown in (C) all statistical analysis were performed using two‐factor anova with Tukey's HSD *post‐hoc* test; letter code indicates statistical differences at *P* < 0.05; n.s., not significant.

We asked how PHR1 might be activated upon short‐term high light. Since PHR1(‐like) activity is primarily regulated by the P_i_ status of the cell, we fractionated plant rosettes subjected to high light and determined subcellular concentrations of free P_i_. Under control conditions, WT and *phr1‐1 phl1* mutant plants clearly differed in their subcellular P_i_ pools (Figure 5A). 20 min of high light treatment caused a decline in chloroplast P_i_ levels in the WT, but not in the double mutant (Figure 5A). We also determined changes upon 5 min of high light exposure in the WT and surprisingly observed a strong increase of plastidial P_i_ levels (Figure S6). Importantly, P_i_ concentrations of the cytosolic/nuclear and vacuolar fractions were not significantly altered by high light in either WT or *phr1‐1 phl1* mutant plants at 20 min of stress (Figure 5A), but increased transiently in the WT at 5 min (Figure S6). Hence, elevated light rapidly induced intracellular P_i_ fluctuations in the WT; however, the effect was mostly confined to the chloroplast and therefore does not qualify as a direct cause of PHR1 activation upon high light via the cytosolic InsP equilibrium.

The metabolism of InsPs depends on both P_i_ and ATP levels (Riemer et al., 2021; Zhu et al., 2019). Given that cytosolic P_i_ concentrations were not significantly reduced by high light treatments, we asked if light increase affected cellular ATP levels. While *phr1‐1 phl1* double mutants exhibited a tendency toward lower ATP levels that was more pronounced upon high light treatment, light increase did not affect ATP or ADP levels in the WT at 20 or 90 min of treatment (Figure 5B). Thus, activation of PHR1 by short‐term high light appears to be independent of ATP levels.

It has been reported that the PSR is modulated by sugar availability (Franco‐Zorrilla et al., 2005; Karthikeyan et al., 2007; Müller et al., 2005, 2007). Since sugars are also involved in the signaling of photosynthetic status (Häusler et al., 2014; Li & Sheen, 2016; Zirngibl et al., 2023), we tested if short‐term high light treatment was already sufficient to induce changes in sugar levels in leaves. Contents of sucrose under control conditions were found to be lower in leaves of *phr1‐1 phl1* as compared with the WT (Figure 5C). Twenty minutes high light treatment resulted in a further increase in sucrose (Figure 5C), but not glucose or fructose (Figure S7) levels in the WT. A similar fold change of sucrose contents was observed in *phr1‐1 phl1* mutant leaves upon high light, although the increase in absolute levels was not significant (Figure 5C). Thus, sucrose levels respond to high light treatment on a very short time scale. In order to test if sucrose might act as a signal during light‐induction of PSRs, we subjected WT rosette leaves to a short sucrose treatment by feeding cut leaves via the petioles with a buffered solution containing either sucrose or an osmotic control for 30 min. While the expression of *SENESCENCE 1* (*SEN1*), also called *DARK‐INDUCIBLE 1* (*DIN1*), a sugar‐repressible gene (Fujiki et al., 2000) was reduced by sucrose feeding in the rosette leaves, *SRG3*, *SPX1*, and *VPE1* did not respond to this treatment (Figure S8), indicating that sucrose is unlikely to cause induction of the PSR during high light.

Together, our results indicate that induction of PSR marker genes upon light increase occurs in at least two phases: In the long term, a shift to higher light intensities entails low‐nutrient signaling caused by cellular P_i_ depletion, likely through an imbalance of growth and nutrient uptake. In contrast, short‐term (20–45 min) responses are triggered by an unknown signal and coincide with fluctuations in subcellular P_i_ pools.

### Carbohydrate accumulation is slightly affected in *phr1‐1 phl1* mutants upon short‐term high light

**Figure 6:**
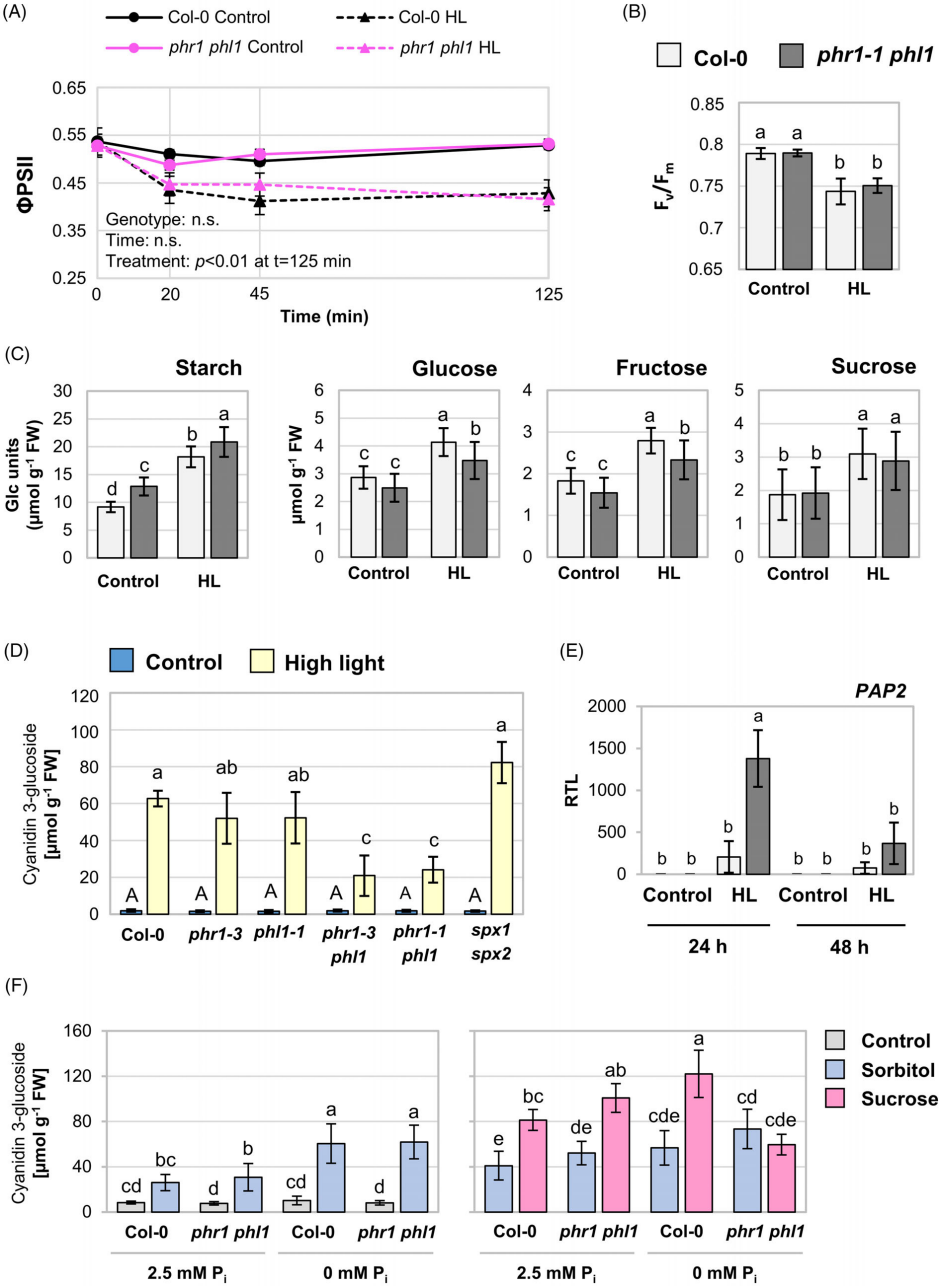
*phr1‐1 phl1* mutants show altered carbohydrate responses. Unless specified, plants were grown and treated as described for Figure 4. Data points and bars depict means ± standard deviations. (A, B) Pulse‐amplitude modulated (PAM) measurements were performed to determine the effect of high light (HL) on chlorophyll a fluorescence parameters. *n* = 3 independent experiments (representing means of each three leaves measured). (A) Quantum yield of photosystem II photochemistry (ΦPSII) in light‐adapted leaves of wild‐type (WT) (Col‐0) and *phr1‐1 phl1* after 20, 45, and 125 min of treatment. (B) Plants were dark‐adapted for 20 min after 125 min of treatment duration to determine the maximum efficiency of photosystem II (*F*_v_/*F*_m_). (C) Changes in carbohydrate levels relative to fresh weight (FW) after 90 min of HL treatment. Starch: *n* = 9 plants from three independent experiments. Glucose, fructose, and sucrose: *n* = 11–12 plants from four independent experiments. (D) Anthocyanin (cyanidin 3‐glucoside) contents upon HL exposure. Plants were grown for 32 days until the onset of the treatments at 4 h into the photoperiod. Leaf material was harvested after an additional 70 h. Pigment contents were calculated relative to FW. *n* = 3 independent experiments. (E) Transcript levels of *PAP2* upon HL. Plants were grown for 39 days before light intensity was shifted to HL at 4 h into the photoperiod. Material was harvested after 24 and 48 h of treatment. Relative transcript levels (RTL) of *PAP2* were calculated as 1000 × 2^−ΔCT^ with *PP2A* as the reference. *n* = 3 independent experiments. (F) *phr1‐1 phl1* mutants are defective in sugar‐induced anthocyanin production under P_i_‐limiting conditions. Seedlings were grown on nutrient‐rich media (no sucrose or sorbitol added) for 7 days before transfer to media with either 0 or 2.5 mM KH_2_PO_4_ added and containing either no additives (control), 90 mM sucrose, or 160.7 mM sorbitol (same osmotic strength). Shoots were harvested for anthocyanin determination after 67 h of growth on differing media. *n* = 6 pools of seedling shoots from three independent experiments. (A, D) One‐way anova with Tukey's HSD follow‐up test and Bonferroni alpha correction for contrasts, *P* < 0.01; n.s., not significant. (D) Statistical analysis was performed individually for each treatment group, indicated by different letter case; different letters indicate statistical differences in genotypes (*P* < 0.01). (B, C, E, F) Letter code indicates statistical differences determined by two‐factor anova with Tukey's HSD *post‐hoc* test (*P* < 0.05).

To address the functional role of PSR induction by high light, we asked if photosynthetic performance was affected by the *phr1‐1 phl1* mutation during the early phase of light acclimation. We first performed pulse‐amplitude modulated (PAM) chlorophyll fluorometry on rosette leaves subjected to increased light. The efficiency of photosystem II photochemistry (ΦPSII) dropped to the same extent in both WT and *phr1‐1 phl1* mutants upon exposure to increased illumination for 125 min (Figure 6A). After 125 min of treatment, also the maximum efficiency of PSII (*F*_v_/*F*_m_) had decreased significantly in the stressed plants of both genotypes (Figure 6B). As for ΦPSII, no difference was observed for *F*_v_/*F*_m_ between WT and *phr1‐1 phl1*, indicating that thylakoid reactions were not affected in *phr1‐1 phl1* mutants. To assess whether carbon fixation was compromised in *phr1‐1 phl1*, we determined the changes in carbohydrate contents that occurred within 90 min upon an increase in photon flux density. As shown in Figure 6C, starch contents under control conditions were higher in *phr1‐1 phl1* mutants than in the WT. This may be related to lower P_i_ levels in the chloroplasts (Figure 5A), a condition that allosterically activates AGPase (Figueroa et al., 2022). High light treatment caused a similar increase in starch contents in both genotypes, accompanied by slightly lower levels of monosaccharides, but not sucrose, in the double mutant (Figure 6C). Notably, differences in sucrose levels that were seen between WT and *phr1‐1 phl1* under control growth at 80 min after the onset of the photoperiod (Figure 5C) were not detected at 150 min into the photoperiod (Figure 6C), indicating that sucrose levels are altered in *phr1‐1 phl1* during the early morning. In conclusion, monosaccharide levels were marginally affected in *phr1‐1 phl1* mutants, while photosynthetic performance and the accumulation of starch and sucrose were normal in the mutant in response to a higher light intensity.

### *phr1 phl1* mutants are impaired in high light‐mediated anthocyanin accumulation

Since both P_i_ starvation and high light stress trigger anthocyanin production, we next analyzed the role of PHR1/PHL1 in anthocyanin accumulation during high light acclimation. As shown in Figure 6D, anthocyanin contents after 3 days of high light exposure were significantly lower in *phr1 phl1* than in WT or the single mutants *phr1‐3* and *phl1*, while loss of *SPX1/2* function did not have any statistically significant effect. PHR1 has been implicated in flavonoid biosynthetic gene induction under low P_i_ (He et al., 2021; Liu et al., 2022). We did not detect significant differences in the induction of *LDOX*, *DFR*, *PAP1*, or *MYB111* transcripts between WT and *phr1‐1 phl1* mutants at 24 or 48 h of light increase (Figure S9). Interestingly, however, *PAP2* expression was markedly upregulated in *phr1‐1 phl1* compared with the WT at 24 h of high light (Figure 6E), indicating that the regulation of anthocyanin accumulation is disturbed at a different level than biosynthetic gene expression in *phr1‐1 phl1*.

It has been reported that high light‐induced anthocyanin biosynthesis depends on sucrose signaling (Zirngibl et al., 2023). Given that, eventually, sucrose levels were similar between WT and *phr1‐1 phl1* upon high light (Figure 6C), we reasoned that high light‐mediated anthocyanin production might be impaired downstream of sucrose accumulation in *phr1‐1 phl1*. To address this, we exposed seedlings to sucrose‐ or sorbitol‐containing media with different P_i_ supplies. Three days of mere P_i_ depletion were not sufficient to trigger measurable anthocyanin production in either genotype (Figure 6F). When sorbitol was added to the media, WT and *phr1‐1 phl1* seedlings accumulated anthocyanins to the same level (Figure 6F). Crucially, in the double mutant, sucrose did not trigger anthocyanins above the level of the osmotic effect when the growth medium lacked P_i_ (Figure 6F). Similar to what was seen under high light (Figure 6D), this effect was not observed in the *phr1‐1* single mutant (Figure S10). In conclusion, sucrose‐mediated anthocyanin production is impaired in *phr1‐1 phl1* double mutants already at early stages of P_i_ depletion, and *PHR1/PHL1* might function redundantly for adequate high light‐mediated anthocyanin accumulation when cellular P_i_ reserves decline in the medium term following light‐induced growth stimulation.

### SRG3 is involved in lipid metabolism upon light increase

**Figure 7:**
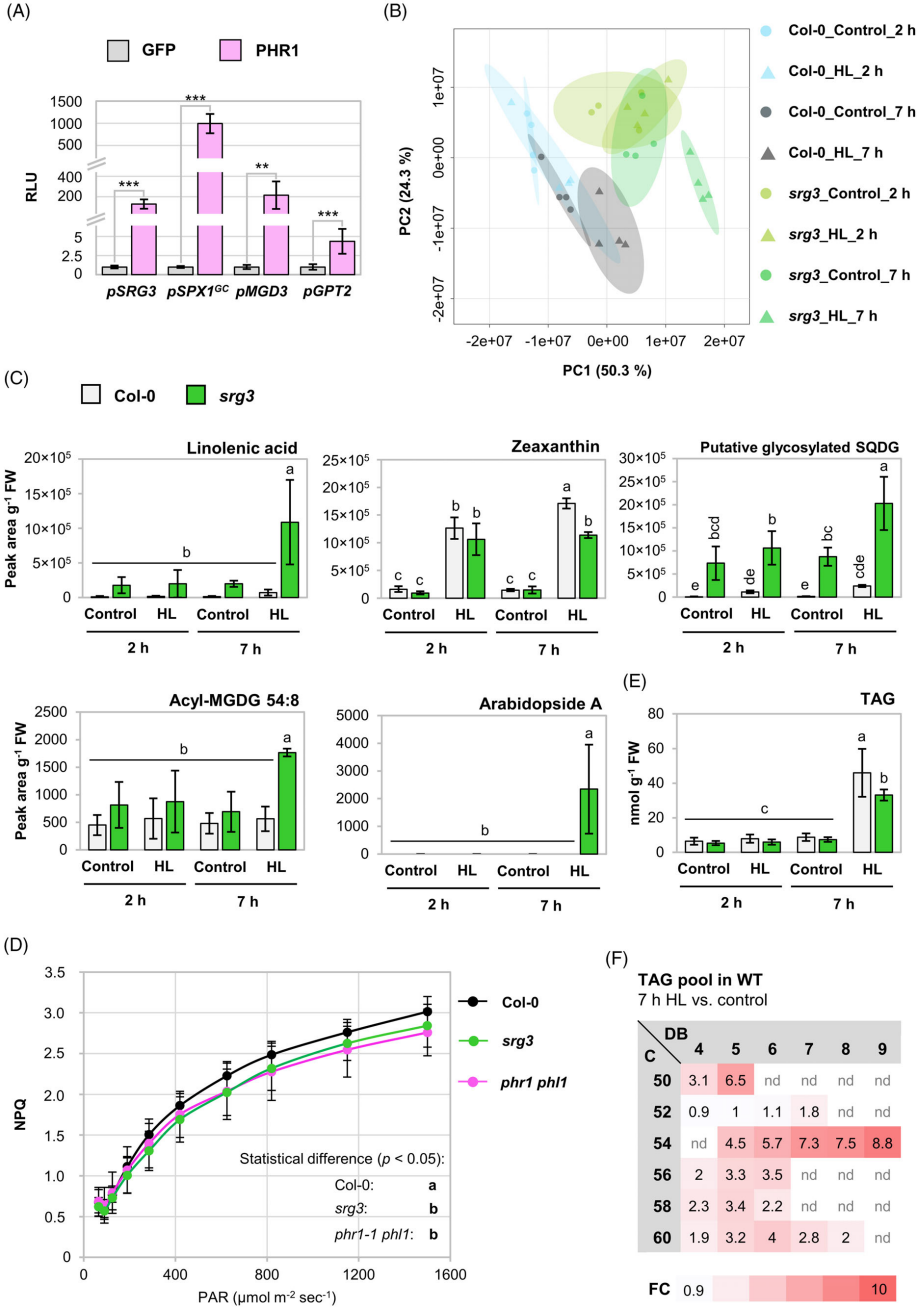
Effect of *srg3* mutation on the lipid composition upon light increase. (A) Promoter–transactivation assay in mesophyll protoplasts. Promoter sequences of *SRG3*, *SPX1* (without *Nco*I site: *pSPX1* ^*GC*^), *MGD3*, and *GPT2* were fused to the firefly *LUC* reporter gene and co‐transfected with either *PHR1* or *GFP*. Luminescence units were normalized to the luminescence of *Renilla LUC* which was additionally co‐expressed from the Cauliflower mosaic virus *35S* promoter, and calculated relative to the luminescence level of *GFP*‐expressing samples. RLU, relative luminescence level. Results for each promoter construct are from independent experiments. *n* = 6 from three independent experiments. One‐way anova with Tukey HSD follow‐up test and Bonferroni alpha correction for contrasts; ***P < 0.001, **P < 0.01. (B, C, E, F) Plants of wild‐type (WT) (Col‐0) and *srg3* genotype were grown and treated as described for Figure 4, but leaves were harvested at 2 and 7 h of treatment. Lipid species were analyzed using LC–MS. (B) Principal component analysis of samples from 2 and 7 h high light (HL)‐treated or control plants. (C) Levels of *srg3*‐dependent HL‐responsive lipid features normalized to leaf fresh weight (FW). *n* = 4 independent experiments, except for acyl‐MGDG: *n* = 3–4. (D) Light response of the non‐photochemical quenching (NPQ) parameter measured by pulse‐amplitude modulated (PAM) chlorophyll fluorometry. Plants of WT, *srg3*, and *phr1‐1 phl1* genotype were grown for 40 days and dark‐adapted for ≥30 min before measurement. NPQ was plotted against the fluence rate of photosynthetically active radiation (PAR). *n* = 3 independent experiments representing means of two plants. (E, F) *n* = 3–4. (E) Contents of triacylglycerols (TAG) in leaves after HL treatments. (F) Changes in TAG pools upon 7 h of light increase (7 h HL versus 7 h control) in the WT. Fold changes (FC, Col‐0_HL_7h/Col‐0_C_7 h) are indicated by color code and were calculated for TAG species sorted by the total number of carbon atoms of the acyl chains (C) and the total number of double bonds (DB); nd, not detected. (C–F) Two‐factor anova with Tukey's HSD *post‐hoc* test; *P* < 0.05. Statistical differences in (C) and (D) are indicated by letter code (*P* < 0.05). All bars are means ± standard deviations.

Among the *PHR1*‐dependent transcripts analyzed under short‐term high light, *SRG3* showed particularly strong upregulation (Figure 4A), and it has been suggested that the *SRG3* promoter is directly activated by PHR1 (Cheng et al., 2011). To test this hypothesis, we performed promoter–transactivation assays in mesophyll protoplasts. PHR1 was able to induce the expression of a luciferase reporter fused to the promoters of *SRG3*, *MGD3*, *GPT2*, and *SPX1* (lacking the naturally occurring *Nco*I restriction site, *proSPX1* ^*GC*^) (Figure 7A). The responsiveness of *proGPT2* to *PHR1* expression was considerably lower, consistent with the observation that *GPT2* was induced independently of *PHR1* under high light (Figure 4A). This suggests that *SRG3*, like *MGD3* and *SPX1*, is a veritable target gene of PHR1.

We next asked whether *SRG3* was relevant for lipid metabolism upon light increase, given the proposed role of SRG3 in phospholipid degradation pathways. Thus, we compared the lipid profiles of 5‐weeks‐old WT and *srg3* loss‐of‐function mutants (Cheng et al., 2011) upon exposure to high light for 2 or 7 h. The 2‐h exposure was an early time point of acclimation, close to the onset of gene expression, while the 7‐h exposure corresponded to the end of the light period to allow for a stronger metabolic response. The first principal component of the PCA analysis (Figure 7B) on the aligned 2478 lipid features (Table S1) accounted for 50% of the total variance and suggested differences in the lipidome of *srg3* compared with the WT. The total levels of the main structural lipids were comparable between the genotypes (Figure S11A), which is consistent with the results obtained for *srg3* mutants under P_i_ starvation conditions (Cheng et al., 2011). Notably, the levels of monogalactosyl diacylglycerol (MGDG) (34:1), digalactosyl diacylglycerol (DGDG) (34:1), and DGDG (34:2) were slightly higher after 7 h of high light in both genotypes (Figure S12A,B), whereas the levels of the phospholipids determined were unchanged (Figure S13). In addition, profiling of sphingolipids revealed non‐significant higher levels of ceramides in *srg3* in all four investigated conditions, but hydroxy ceramides and glucosyl ceramides were WT‐like (Figure S11B).

We also performed profiling of acyl‐MGDGs and Arabidopsides in the extracts, as galactolipids can be acylated or oxygenated (Ibrahim et al., 2011). We could only identify acyl‐MGDG‐54:8 and Arabidopside A (1‐OPDA, 2‐dnOPDA MGDG), and their levels were significantly higher in *srg3* after 7 h of high light stress compared with the other groups (Figure 7C). To identify more *srg3*‐dependent high light‐responsive lipids, we performed anova and first selected 81 lipid features that were significantly different in WT plants upon 7 h of high light (fold change ≥2, *P* ≤ 0.05; Table S2). Among those, 18 were significantly different in *srg3* compared with WT after 7 h of high light (Table S3). One of them was identified as linolenic acid, the levels of which were higher in *srg3* than in WT after 7 h of high light (Figure 7C), like Arabidopside A and acyl‐MGDG‐54:8. Interestingly, a lipid feature was constitutively higher in *srg3* and accumulated upon high light treatment (Figure 7C; Figure S14) which we could tentatively assign to a glycosylated SQDG species (SQDG‐34:1+C_5_H_8_O_4_; Table S3). The identification of this unknown lipid species will need to be verified in the future.

One of the *srg3*‐dependent metabolite features showing highest light‐responsiveness was identified as zeaxanthin (Figure 7C; Figure S14; Table S3), a photoprotective carotenoid and component of the xanthophyll cycle (Demmig‐Adams & Adams, 1996; Havaux & Niyogi, 1999). In *srg3*, zeaxanthin accumulation following 7 h of high light was reduced, indicative of a constrained capacity for light energy dissipation in chloroplast membranes of *srg3*. To further investigate this, we performed PAM chlorophyll fluorescence measurements and determined the light response of the non‐photochemical quenching (NPQ) parameter in WT, *srg3*, and *phr1‐1 phl1* mutant plants. Indeed, the extent of NPQ was slightly but significantly reduced in both the *srg3* and the *phr1‐1 phl1* mutants compared with the WT (Figure 7D). Taken together, *SRG3* expression is required to control linolenic acid levels and for the establishment of light protection upon high light stress.

### *SRG3* contributes to triacylglycerol (TAG) accumulation upon light increase

Linolenic acid accumulation in *srg3* leaves upon high light (Figure 7C) raised the question whether containment of free fatty acids was impaired by *srg3* mutation. One strategy to reduce the levels of cytotoxic fatty acids in plant cells is their incorporation into TAG under stress, including P_i_ starvation (Pant et al., 2015; Yang & Benning, 2018). We therefore quantified total TAG levels and found a pronounced accumulation after 7 h of light treatment in the WT which was significantly lower in *srg3* mutants (Figure 7E). The difference was due to reduced accumulation of TAG species with long‐chain polyunsaturated fatty acids exhibiting a higher degree of desaturation (54:6, 54:7, 54:8, 54:9) in *srg3* (Figure S15), which would be accordant with linolenic acid esterification. TAG species with polyunsaturated 54‐carbon acyl chains also showed the highest changes in the WT upon high light (Figure 7F), a pattern that is reminiscent of heat‐stress‐induced TAG accumulation (Mueller et al., 2015). To control if high light caused a heat response in the treated plants, we analyzed transcript levels of *HEAT SHOCK PROTEIN 70* (*HSP70*) and *HSP18.2*, previously used to discriminate light and heat responses (Huang et al., 2019). High light caused accumulation of *HSP70* and, to a lesser extent, *HSP18.2* transcripts which was mostly confined to the 45 min time point of treatment (Figure S16A). While *HSP70* induction upon high light was also seen by Huang et al. (2019), *HSP18.2* transcript accumulation is indicative of a mild heat response under the conditions used in this study. Thus, to test if *SRG3*‐dependent TAG accumulation was triggered by higher temperature at the leaf surface upon high light, we assessed *SRG3* gene expression after a TAG‐inducing heat treatment of 38°C–40°C (Mueller et al., 2015). While *HSP18.2* and *HSP70* responded massively, *SRG3* transcript levels remained very low during heat treatments (Figure S16B). Hence, our analysis of TAG accumulation in *srg3* mutant plants suggests that *SRG3* gene function is required for normal occurrence of high light‐induced TAG enrichment, and this effect is independent of any radiation‐mediated temperature increase.

## DISCUSSION

**Figure 8:**
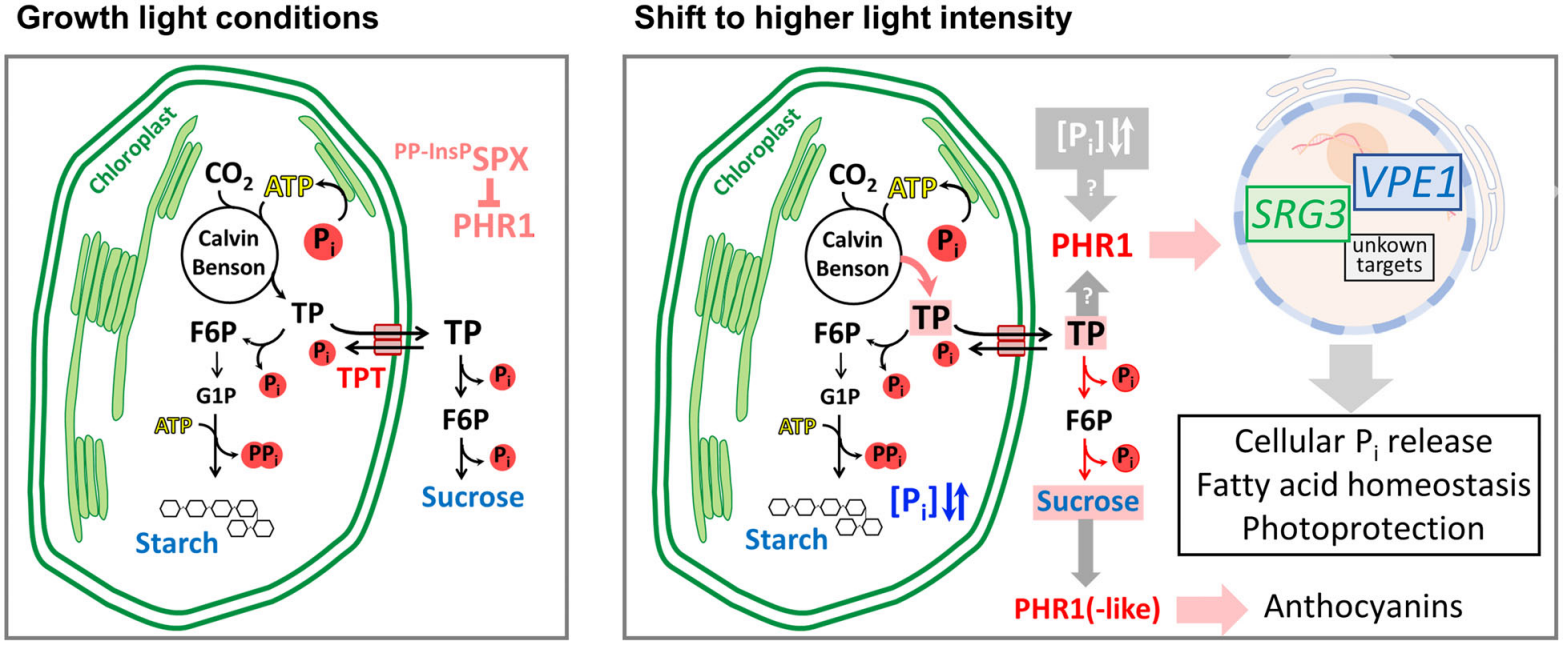
Model of P_i_ signaling under light increase. (Left) Under normal growth light conditions, production of triose phosphates in the Calvin–Benson cycle and their utilization in downstream processes such as starch and sucrose biosynthesis are balanced, and the rate of P_i_ recycling matches its consumption by the chloroplast metabolism (McClain & Sharkey, 2019; Stitt et al., 1987). PHR1 is kept inactive in the InsP–SPX complex. Right, Upon shift to higher fluence rates, phosphorylated photosynthetic intermediates accumulate (Borghi et al., 2019), and chloroplast P_i_ concentrations fluctuate. Chloroplast P_i_ levels, a transient drop in cytosolic P_i_ concentrations, or changes in the pools of photosynthetic products might generate a signal that leads to PHR1 activation. This causes PSR gene expression including the induction of *SRG3* and *VPE1*, leading to P_i_ release from intracellular pools and changes in lipid metabolism enabling the containment of free fatty acids and xanthophyll cycle operation. In the medium term, increments in sucrose levels trigger anthocyanin accumulation which is supported by PHR1‐like proteins when cellular P_i_ reserves decline upon growth stimulation. F6P, fructose 6‐phosphate; G1P, glucose 1‐phosphate; TP, triose phosphate.

P_i_ is intimately linked to photosynthetic metabolism, both as a substrate for photophosphorylation and as a kinetic regulator of carbon assimilation (Furbank & Lilley, 1980; Hendriks et al., 2003; Marcus et al., 2005; Preiss, 1984; Stitt et al., 1983). Additionally, PSRs affect the photosynthetic machinery of plants (Fredeen et al., 1990; Giersch & Robinson, 1987; Liu et al., 2024), which is partially achieved at the transcriptional level (Bustos et al., 2010; Morcuende et al., 2007; Nilsson et al., 2012; Wu et al., 2003). The particular role of phosphorus for global primary production was recently demonstrated (Wang et al., 2025). Nevertheless, it has remained elusive whether changes in the metabolic P_i_ demand of the chloroplast relay to the nuclear PSR. The observations presented herein establish that the P_i_ response machinery involving the transcription factor PHR1 is activated upon an increase in photosynthetic activity, and we provide evidence that PHR1 contributes to the rapid metabolic adjustment during changes in light intensity (Figure 8).

### Increase in light intensity creates a transient low‐P_i_ signal

We noted that the transcriptomic changes upon a shift to high light overlap with those observed during growth under P_i_‐limited conditions (Figure 3A). Interestingly, a previous study addressing intraspecies variation of the transcriptional response to increased irradiance recognized the induction of PSR genes upon high light in two out of three tested accessions of Arabidopsis, and PHR1‐target gene induction coincided with photosynthetic efficiency of the ecotypes (van Rooijen et al., 2018). Among the high light and low‐P_i_ induced transcripts, *SRG3*, *SPX1*, and *VPE1* accumulated transiently during the early phase of light acclimation (Figure 4). Crucially, gene induction was absent in *phr1* mutants, qualifying this short‐term high light response as dependent on nuclear low‐P_i_ signaling. Hence, plant cells are capable of exploiting the transcriptional potential of the low‐P_i_ response module in order to react to environmental stimuli, even under nutrient‐sufficient conditions. Notably, high light‐mediated induction was not seen to the same extent for all PHR1 targets examined (Figure 4A), suggesting that short‐term high light affects only a subset of the transcriptional PSR, potentially reflecting differential chromatin accessibility of the genes (Barragán‐Rosillo et al., 2021).

How does high light affect subcellular P_i_ levels? It has been established that photosynthetic activity, through the production of phosphorylated assimilates, reduces the P_i_ pool available for photophosphorylation (McClain & Sharkey, 2019; Robinson & Giersch, 1987; Sharkey, 1985; Walker & Sivak, 1986). Indeed, our analysis of subcellular P_i_ contents revealed strong fluctuations of chloroplast P_i_ upon an increase in light intensity, but not in the cytosolic/nuclear or the vacuolar fractions (Figure 5A; Figure S6). This finding is consistent with the assumption that P_i_ exchange between chloroplast and cytosol (Santarius & Heber, 1965), and even more so between cytosol and vacuole (Woodrow et al., 1984), are rather slow processes. Additionally, the rapid increase in sucrose levels that can be observed upon high light (Figure 5C) indicates that the capacity for P_i_ liberation via sucrose biosynthesis might be sufficient to balance cytosolic P_i_ levels against depletion through triose phosphate import.

These findings raise the question of how PHR1 is activated upon high light. Given the large difference in vacuolar P_i_ levels compared with the cytoplasm, we cannot fully rule out that a difference in the P_i_ levels between treated and control cytosolic preparations was masked by minor vacuolar contaminations. It is also possible that a drop in cytosolic P_i_ (and hence InsP) levels sufficient to trigger a peak in PHR1 activity occurs transiently and was not captured by our analysis. In fact, reduced cytosolic/nuclear P_i_ pools accompanied by constitutive PSR gene expression were observed in a less volatile experimental setup when triose phosphate utilization pathways were perturbed by genetic manipulation in the *adg1 tpt‐1* genotype (Figure 2B,C), which is coherent with InsP‐dependent PHR1 activation. In contrast, vacuolar P_i_ pools in *adg1 tpt‐2* mutants were high (Figure 2C), potentially reflecting both activated P_i_ acquisition and a high capacity for P_i_ recycling through vacuolar phosphatase activity acting on phosphorylated organic compounds such as phosphoenolpyruvate (Ohnishi et al., 2018). Interestingly, *adg1 tpt‐2* also exhibited increased plastidial P_i_ pools, similar to what was seen upon high light at a very early timepoint (Figure S6). It is unclear why plastidial P_i_ pools increase under both conditions, or whether fluctuations in chloroplast P_i_ relate to nuclear P_i_ signaling. Hence, future studies are required to investigate both InsP levels upon light increase, as well as the intriguing possibility that chloroplast P_i_ levels act in retrograde signaling.

### Sugar responses are altered in *phr1 phl1* mutants

Slow growth and developmental delay of the *adg1 phr1 phl1* triple mutant (Figure 1B–D; Figure S1C) indicate that starch biosynthesis aids metabolism when cellular P_i_ levels are chronically depleted. This finding supports the conclusion that proper triose phosphate recycling is crucial for cellular P_i_ availability. The fact that growth restriction of *phr1‐3 phl1* double and *adg1 phr1 phl1* triple mutants under high light was alleviated by sucrose in the growth medium (Figure 1D) further indicates that these mutants suffer from an energetic deficit. This is also reflected by slightly reduced ATP levels in *phr1 phl1* plants at the adult state (Figure 5B). Interestingly, *phr1 phl1* seedlings were also insensitive toward sucrose in terms of anthocyanin production under early P_i_‐depleted growth conditions (Figure 6F), and anthocyanin accumulation upon high light was reduced despite regular sucrose levels in adult plants (Figure 6C,D). In contrast to the short‐term light response, these phenotypes were not seen in *phr1* single mutants (Figure 6D; Figure S10). Hence, PHR1‐like proteins might be redundantly involved in the downstream pathways of sugar signaling, including anthocyanin production in the nutrient‐limited long‐term phase of light acclimation (Figure 3).

### PHR1 promotes acclimation to higher light intensities

Our analysis of the *PHR1‐*dependent transcriptional response to high light focused on *SRG3*, *SPX1*, and *VPE1*. *VPE1* putatively encodes a vacuolar efflux transporter (Xu et al., 2019), suggesting a strategy for intracellular P_i_ redistribution in favor of the cytosol by exploitation of the vacuolar P_i_ stores. This would serve to provide additional P_i_ to metabolic pathways when light increase accelerates the growth rate, and *VPE1* gene induction is consistent with declining cellular P_i_ reserves upon high light in the long term (Figure 3B). In contrast, *SPX1*, as a highly responsive PSR marker gene (Wang et al., 2023), functions in fine‐tuning of PSRs (Puga et al., 2014; Whitfield et al., 2026). This seems appropriate given the fluctuating nature of light as an environmental factor.

The mechanistic contribution of SRG3 to P_i_ homeostasis is indistinct, and it has been reported in other plant species that GDPD isoforms not only play a role in phospholipid degradation (Dang et al., 2025), but also in hormone signaling and the modulation of root morphology upon P_i_ starvation (Hu et al., 2024; Mai et al., 2021; Verma et al., 2025). Strikingly, we detected differences in the lipid fingerprints of WT and *srg3* particularly after a longer duration of light treatment (Figure 7B). Among the molecules that overaccumulated in *srg3* mutants in a high light‐dependent manner, we identified linolenic acid, the galactolipid derivatives acyl‐MGDG (54:8), and Arabidopside A, as well as a molecule that was constitutively upregulated in *srg3* and putatively represents glycosylated SQDG (Figure 7C). Both acyl‐MGDG and Arabidopside accumulation have been reported to be associated with stress conditions (Buseman et al., 2006; Nilsson et al., 2016; Vu et al., 2014), and it was suggested that the pool of acyl‐MGDG increases upon stress to sequester oxidized fatty acids (Vu et al., 2014). Moreover, Arabidopside A has been ascribed a role in the regulation of chlorophyll degradation during senescence processes (Hisamatsu et al., 2006; Weber, 2002). Thus, *SRG3* induction might be required to maintain the integrity of photosynthetic membrane components upon changes in light availability. Accumulation of linolenic acid in leaves of light‐stressed *srg3* plants furthermore indicates that the processing of fatty acids is disturbed in the mutant. The product of SRG3 activity, glycerol‐3‐phosphate (Cheng et al., 2011), is a substrate for membrane lipid biosynthesis, as well as for TAG production (Bates, 2016). We detected accumulation of TAGs containing linolenic acid in the WT after 7 h of light increase (Figure 7E,F; Figure S15). Consistent with a potential involvement of SRG3 in fatty acid containment, TAG accumulation was slightly reduced in *srg3*. Elevated levels of free fatty acids can be cytotoxic probably due to their amphipathic and detergent‐like properties leading to the inhibition of photosynthesis and damage to PSII (Kunz et al., 2009). Thus, our results suggest that SRG3 contributes to the detoxification of free linolenic acid, potentially by enabling incorporation of excess linolenic acid into TAGs. Notably, linolenic acid accumulation was previously also observed in Arabidopsis plants subjected to salinity stress, and this was connected to the control of ion homeostasis via the regulation of H^+^‐ATPase activity (Abdelrahman et al., 2021; Han et al., 2017). Hence, instead of a direct involvement of SRG3 in the processing of fatty acids, linolenic acid accumulation in *srg3* might also be a secondary effect related to perturbed membrane remodeling.

Interestingly, after 7 h of high light, we observed an increase in galactolipid species with low degree of desaturation (34:1; 34:2) in WT and mutant plants (Figure S12A,B), an aspect of short‐term high light acclimation that has been reported before (Burgos et al., 2011). MGD1, the enzyme predominantly responsible for the initiation of galactolipid biosynthesis in P_i_‐sufficient conditions (Jarvis et al., 2000; Kobayashi et al., 2007), draws on 34:1‐DAG precursors derived from both plastidial (18:1, 16:0) and endoplasmic (16:0, 18:1/18:2) origin, and galactolipids are subsequently desaturated (Hölzl & Dörmann, 2019; Ohlrogge & Browse, 1995). Hence, low desaturation indicates the accumulation of early products of galactolipid biosynthesis. In fact, fatty acid biosynthesis in the plastid is activated by light through a redox cascade (Sasaki et al., 1997), and it was hypothesized by Burgos et al. (2011) that NADPH‐stimulated fatty acid biosynthesis might exceed the capacity for fatty acid desaturation under high light. Moreover, TAG accumulation confirms high lipid turnover upon high light. Notably, an increase in the contents of fatty acids with low degree of saturation (16:0, 16:1, 18:0, 18:2) in MGDG and DGDG fractions was seen also under P_i_‐starved conditions, and this was ascribed to the action of the P_i_ starvation‐induced galactolipid synthases (Kelly et al., 2003; Kobayashi et al., 2004). Although *MGD3* induction was mild under the conditions used in this study (Figure 4A), galactolipid biosynthesis might be accelerated under high light by the combined activities of type A (MGD1) and type B (MGD2/3) enzymes. Whether this aids in the maintenance of membrane integrity during high light acclimation will be an interesting question to address during future studies.

A compound that accumulated upon light increase in the WT, but was reduced by *srg3* mutation, was identified as zeaxanthin (Figure 7C), a molecule that is involved in the protection against excess excitation energy as part of the xanthophyll cycle (Demmig‐Adams & Adams, 1996; Havaux & Niyogi, 1999; Sacharz et al., 2017). In line with a function of both *SRG3* and *PHR1*/*PHL1* for proper operation of the xanthophyll cycle, assessment of the NPQ component of chlorophyll a fluorescence revealed deficits in both mutant genotypes (Figure 7D). It is well known that the membrane lipid composition is important for the xanthophyll cycle, and both lipid class and fatty acid composition determine faithful operation of all components (Goss & Latowski, 2020). Hence, it is tempting to hypothesize that *SRG3* expression, induced by the nuclear low P_i_ response machinery, might be required for the adjustment of the membrane environment to ensure proper function of the enzymes involved in the xanthophyll cycle. Intriguingly, this might be directly related to fluctuations in chloroplast P_i_ levels seen under high light (Figure 5A; Figure S6), given the recent observations on P_i_‐transport mutants made by Raju et al. (2024). Here, P_i_ levels in the stroma correlated with ΦPSII and NPQ parameters. We therefore propose a signaling pathway originating from photosynthetic activity that engages PHR1 to induce *SRG3* expression for the acclimation of the photosynthetic membranes toward high light.

## CONCLUSIONS

High light stress requires immediate responses to avert damage to cellular components. As a new mode of signaling excess photosynthetic activity, we describe a function of the low‐P_i_ response machinery involving the transcription factor PHR1 under nutrient replete growth conditions. Our results indicate that changes in subcellular P_i_ concentrations and/or the accumulation of photoassimilates create a retrograde signal resulting in the activation of PHR1 in the cytosol and nucleus. This serves to mobilize cellular P_i_ and to support acclimation responses including the adjustment of lipid metabolism to ensure photoprotection and free fatty acid containment. In the long term of light acclimation, PHR1 and (‐like) proteins are furthermore engaged in anthocyanin production upon growth‐induced cellular P_i_ restriction.

## MATERIALS AND METHODS

### Plant material

All Arabidopsis plants used in this study are in the background of the Columbia‐0 accession. The *phr1‐3* (AT4G28610; SALK_067629C) (Nilsson et al., 2007) and *phl1* (AT5G29000; SAIL_731_B09) (Klecker et al., 2014) lines were described previously and the derived homozygous double mutant *phr1‐3 phl1* was a kind gift of Angelika Mustroph (University of Bayreuth). The double mutant *phr1‐1 phl1* (Bustos et al., 2010) was backcrossed to *phl1* in order to remove a transgene containing the *NPTII* cassette which was previously introduced for isolation of the *phr1‐1* allele (Rubio et al., 2001). All experiments were performed with the homozygous double mutant *phr1‐1 phl1* lacking the *NPTII* cassette. This line was furthermore backcrossed to Col‐0 to obtain the *phr1‐1* single mutant. The double mutant *spx1 spx2* was described earlier (Puga et al., 2014). Knockout lines *adg1‐1* (At5g48300) (Lin et al., 1988), *tpt‐2* (At5g46110; SALK_073707.54.25.x) and *adg1 tpt‐2* (Schmitz et al., 2012) were kindly provided by Rainer E. Häusler (University of Cologne). *gpt2‐1* (AT1G61800; GK‐454H06) and *srg3*/*gdpd1‐1* (AT3G02040; SALK_087106) were obtained from The Nottingham Arabidopsis Stock Centre (NASC) and were described previously (Cheng et al., 2011; Niewiadomski et al., 2005). Primer sequences used for genotyping are listed in Table S4. Homozygous insertion mutants were verified using the following primer combinations: *PHR1* WT: PHR1_F/PHR1_R; *phr1‐3* T‐DNA: LBb1/PHR1_R; *PHL1* WT: PHL1_F/PHL1_R; *phl1* T‐DNA: PHL1_F/LB3; *TPT* WT: TPT_F/TPT_R; *tpt‐2* T‐DNA: *tpt‐2*_F/LBb1; *GPT2* WT: GPT2_F/GPT2_R; *gpt2‐1* T‐DNA: GK‐LB/GPT2_R; *SRG3* WT: qSRG3_F/SRG3_R; *srg3* T‐DNA: qSRG3_F/LBb1. To generate the *adg1 phr1 phl1* triple mutant, *adg1‐1* was crossed to *phr1‐3 phl1*. Homozygous *adg1‐1* mutants were identified by screening for starch‐free phenotypes by iodine staining at the end of the photoperiod. Homozygosity of the *phr1‐1* allele was verified by Sanger sequencing (Macrogen Europe, Amsterdam, The Netherlands) of the PCR product of PHR1_F/PHR1_R using primer PHR1_R.

### Cultivation conditions

Seeds were stratified for 64 h at 4°C in the dark. For vegetative growth, plants were germinated and grown in an 8 h/16 h (light/dark) cycle and 23°C/22°C day/night temperature on soil consisting of seeding compost, universal soil, and vermiculite mixed in a 3:3:1 ratio. For high light experiments, the plants were additionally fertilized once directly after pricking at the age of 10 days using a complete mineral mixture (Wuxal Super, Aglukon Spezialduenger, Duesseldorf, Germany) according to the manufacturer's instructions. For seedling experiments, seeds were surface sterilized by chlorine gas exposure and transferred to solidified Arabidopsis growth media as described in the figure legends. Seedlings were grown under 16 h/8 h light/dark cycles at 23°C. If not indicated otherwise, growth light was set to 90 ± 10 μmol m^−2^ sec^−1^ (for seedling growth and for experiments shown in Figures 1B,C and 2), or 70 ± 5 μmol m^−2^ sec^−1^ (before high light treatments at 450 ± 30 μmol m^−2^ sec^−1^ of rosette plants), and monitored using an illuminance meter. Growth and treatments of rosette plants for high light experiments were conducted in a Percival SE‐41L plant growth incubator equipped with dimmable fluorescent lamps under temperature control.

### P_i_ limitation experiments

For P_i_ limitation experiments, seeds were germinated on medium containing MS salts at full strength together with 0.8% (w/v) phytoagar (Duchefa Biochemie, Haarlem, The Netherlands) and 0.5% (w/v) sucrose. After 5–10 days of growth (see figure legends), seedlings were transferred to P_i_ media (0.5% (w/v) sucrose; 20 mM 2‐(N‐Morpholino)‐ethane sulphonic acid; 2.5 mM KNO_3_; 1 mM MgSO_4_; 1 mM Ca(NO_3_)_2_; 2.5 mM KH_2_PO_4_; 25 μM Fe‐EDTA; 35 μM H_3_BO_3_; 7 μM MnCl_2_; 0.25 μM CuSO_4_; 0.5 μM ZNSO_4_; 0.1 μM NaMoO_4_; 5 μM NaCl; 0.005 μM CoCl_2_; pH 6) solidified by 0.8% (w/v) agar (adopted from Härtel et al. (2000)). For P_i_ depleting conditions, KH_2_PO_4_ was omitted, and Fe‐EDTA was reduced to 10 μM in order to minimize low P_i_‐induced iron toxicity (Ward et al., 2008). Seedling shoots were harvested after 7–8 days of growth on P_i_‐differing media (see figure legends). For the experiment shown in Figures 1D and Figure S2, P_i_ medium was used containing either 0.25 mM KH_2_PO_4_/10 μM Fe‐EDTA, 2.5 mM KH_2_PO_4_/25 μM Fe‐EDTA, or 2.5 mM KH_2_PO_4_/25 μM Fe‐EDTA +50 mM sucrose. For the experiment shown in Figure 6F and Figure S10, seedlings were germinated on MS salts at ½ strength without added sucrose and grown for 7 days before transfer to P_i_‐differing conditions.

### Vector construction

Standard molecular cloning procedures were applied. Primer sequences used for cloning are listed in Table S4. The firefly *LUCIFERASE* reporter plasmid *pBT10‐proMGD3::LUC*_*Firefly*_ and the *pHBTL‐p35S::3HA‐GFP* effector plasmid (Klecker et al., 2014), as well as the *pBT10‐p35S::LUC*_*Renilla*_ normalization plasmid (Bäumler et al., 2019) have been described elsewhere. For details on cloning *proSPX1* ^*GC*^, *proSRG3*, *proMGD3*, *proGPT2*, the *PHR1* effector construct, and *proPHR1::3xHA‐genomicPHR1* for the generation of stably transformed *phr1‐1 phl1* plants, refer to the Supporting Information—Methods.

### Anthocyanin determination

Anthocyanin contents were determined with few changes as described in Vandenbussche et al. (2007). In brief, 40–70 mg of seedling shoots or rosette leaf material were frozen in liquid nitrogen and ground in a bead mill (MM400, Retsch, Haan, Germany). The frozen powder was resuspended in 300 μl of methanol containing 1% HCl. After vortex mixing for 15 sec, cell debris was pelleted by centrifugation for 15 min at >14 000 ***g***, 4°C. The supernatant was mixed with 200 μl water and 500 μl chloroform by vortexing for 15 sec. After 15 min centrifugation at 17 900 ***g***, 4°C, the upper phase was diluted in methanol to measure the absorbance at 535 and 630 nm using a spectrophotometer (Specord 200 Plus, Analytik Jena, Jena, Germany).

### Measurements of starch, soluble sugars, and adenylates

Carbohydrates were determined by the Warburg optical test and adenylates were assessed based on luciferase activity. Both procedures were described previously (Mustroph et al., 2006), for details see Supporting Information—Methods.

### Determination of P_i_ contents

Free P_i_ was determined in leaf tissue based on the method described by Ames (1966) with some modifications partially based on Sakuraba et al. (2018). For the experiment shown in Figure 3B, leaf material was lyophilized prior to extraction, and 50 μl extraction buffer per mg plant material was used. For details see Supporting Information—Methods.

### Non‐aqueous fractionation of leaf material

For the analysis of subcellular P_i_ distribution, 12 plant rosettes (24 rosettes of *adg1‐1*, ~110 of *adg1 tpt‐2*) were harvested into vials precooled by liquid nitrogen and dry ice, with an averaged harvesting time of 3 sec (max. 7 sec) per rosette. The plant material was ground to a fine powder and lyophilized. The freeze‐dried powder was suspended in a mixture of tetrachlorethylene and n‐heptane and fractionated across a non‐aqueous density gradient as described before (Hernandez et al., 2023). Following fractionation, marker enzyme activities for plastids (alkaline pyrophosphatase), cytosol (UDP‐glucose pyrophosphorylase), and vacuole (acidic phosphatase) were determined photometrically. Phosphate amounts were determined as described above for each fraction and correlated with the marker enzyme activities using the NAFalyzer app (https://github.com/cellbiomaths/NAFalyzer).

### RNA extraction and cDNA synthesis

Plant material was harvested under the respective experimental conditions and instantly frozen in liquid nitrogen. The tissue was ground in a bead mill, and RNA was extracted using Bioline TRIsure (Meridian Bioscience, Cincinnati, OH, USA) according to the manufacturer's instructions. The quality of the RNA was assessed by screening *A*_260_ and *A*_280_. For qRT‐PCR analysis, 1 μg of RNA was treated with DNAse I (Thermo Fisher Scientific, Waltham, MA, USA) according to product instructions before application in reverse transcription using oligo(dT)_15_ and RevertAid Reverse Transcriptase (Thermo Fisher Scientific). Complementary DNA was diluted by a factor of 50 for use as template in qPCR analysis. qPCR was performed in technical triplicates using SsoAdvanced Universal SYBR Green Supermix (Bio‐Rad, Hercules, CA, USA) on a CFX Connect Real‐time System (Bio‐Rad). Transcript levels were normalized to the levels of *PP2A* transcript and calculated as 1000 × 2^−ΔCT^. Primer sequences are listed in Table S4.

### Protoplast isolation, transfection, and transactivation assay

Leaf mesophyll protoplasts were isolated using the ‘Tape‐Arabidopsis Sandwich’ method (Wu et al., 2009) with minor modifications. For details, see Supporting Information—Methods.

### Chlorophyll fluorescence measurements

For the determination of the quantum yield of photosystem II photochemistry (*F*_m_′ − *F*′)/*F*_m_′ (ΦPSII), as well as the maximum efficiency of photosystem II (*F*_v_′/*F*_m_′), and the NPQ parameter (or SV_N_; calculated by the Junior PAM software using the Stern–Volmer equation for fluorescence quenching; Gilmore & Yamamoto, 1991), PAM fluorescence measurements of chlorophyll fluorescence were conducted using a Junior PAM (Walz, Effeltrich, Germany). Here, to calculate chlorophyll fluorescence parameters using the saturating pulse method (Schreiber et al., 1986), one large rosette leaf was held between two magnets and subjected to 10 sec of actinic light irradiance set to control light intensity (70 ± 5 μmol m^−2^ sec^−1^), followed by a saturation light pulse.

### Lipid analysis

Total lipid extracts of shock‐frozen leaf material were analyzed by ultra‐performance liquid chromatography coupled to a quadrupole time‐of‐flight mass spectrometer equipped with electrospray ionization source, according to Mueller et al. (2015). Progenesis QI (version 2.1, Waters, Milford, MA, USA) was used for data pre‐processing and PCA was performed in the MetaboAnalyst platform (version 6.0, www.metaboanalyst.ca).

### Western blot analysis

Rosette leaves were harvested into liquid nitrogen, ground to powder in a bead mill, and extracted using 4 μl mg^−1^ fresh weight of 2× Laemmli sample buffer (120 mM TRIS·HCl, pH 6.8; 20% glycerol; 4% SDS; 10% β‐mercaptoethanol). Boiled extracts were separated using 10% acrylamide SDS‐PAGE, and PVDF membranes were decorated with anti‐HA primary antibody (H9658; Sigma‐Aldrich, St. Louis, MO, USA; 1:3000) and secondary antibody (anti‐mouse, A9044; Sigma‐Aldrich; 1:10 000) coupled to HRP. Signals were detected using an enhanced chemiluminescence kit (Amersham^TM^, #RPM2235, Cytiva, Marlborough, MA, USA).

### Statistical analysis

Statistical analyses were performed with Microsoft Office Excel using the Real Statistics Resource Pack software (www.real‐statistics.com). 2‐factor anova and hypergeometric testing were performed using RStudio (version 2024.09.1+394) available at https://www.r‐project.org/.

## AUTHOR CONTRIBUTIONS

MK, conceptualization; AF, TN, and MK, methodology; LA, AF, TN, and MK, formal analysis; LA, AF, LS, TN, AJH, MM, and MK, investigation; MK, writing—original draft; all authors, writing—review and editing; AF and MK, visualization; MJM and MK, supervision; MK, project administration.

## CONFLICT OF INTEREST

None declared.

## Supporting information


**Figure S1.** Phenotypes of *adg1‐1 phr1‐3 phl1* (TM).
**Figure S2.** Root growth of *adg1 phr1 phl1* and *adg1 tpt‐2* responds to exogenously applied sucrose.
**Figure S3.** Expression of selected P_i_ starvation‐responsive genes depends on *PHR1/PHL1* under P_i_ depletion.
**Figure S4.** Changes in sugar and starch contents under P_i_ depletion.
**Figure S5.** *PHR1* transcript or protein levels are not affected by high light exposure.
**Figure S6.** Changes in subcellular P_i_ pool sizes upon short‐term high light.
**Figure S7.** Monosaccharide levels after 20 min of high light.
**Figure S8.** PSR marker genes are not induced in rosette leaves by sugar feeding.
**Figure S9.** Anthocyanin biosynthetic and regulatory gene expression upon high light (HL).
**Figure S10.** Anthocyanin production in response to sucrose is normal in the *phr1‐1* single mutant.
**Figure S11.** Total levels of lipid classes in WT (Col‐0) and *srg3* mutants upon shift to high light (HL).
**Figure S12.** Contents of glycosylglycerol lipid species in WT (Col‐0) and *srg3* mutants upon shift to high light (HL) relative to fresh weight (FW).
**Figure S13.** Contents of glycerophospholipid species in WT (Col‐0) and *srg3* mutants upon shift to high light (HL) relative to fresh weight (FW).
**Figure S14.** Levels of zeaxanthin and putative glycosylated SQDG determined in opposite ESI ion mode compared with Figure 7C.
**Figure S15.** Levels of triacylglycerol (TAG) species in WT (Col‐0) and *srg3* mutants upon shift to high light (HL) relative to fresh weight (FW).
**Figure S16.** *SRG3* gene expression upon light increase is not part of a heat response.

**Table S1.** Annotated lipid features in WT (Col‐0) and *srg3* mutants following a shift to high light.
**Table S2.** Lipid features whose levels were significantly altered upon shifting to the WT (Col‐0).
**Table S3.** Heat‐responsive lipid features whose levels differed significantly between WT (Col‐0) and *srg3* mutants.

**Table S4.** List of primer sequences used in this study.

**Data S1.** Supplemental methods and references.

## Data Availability

The source data of lipid analyses are available in the Supporting Information of this article.
