# Supplementary material for: PHR1 mediates rapid high light responses and acclimation to high photosynthetic activity
Source: Plant J. 2026 May 5;126(3):e70901. doi: 10.1111/tpj.70901 (PMC13139890; doi:10.1111/tpj.70901)
Supplement: Supplementary file 1 — Figure S1. Phenotypes of adg1‐1 phr1‐3 phl1 (TM). Figure S2. Root growth of adg1 phr1 phl1 and adg1 tpt‐2 responds to exogenously applied sucrose. Figure S3. Expression of selected Pi starvation‐responsive genes depends on PHR1/PHL1 under Pi depletion. Figure S4. Changes in sugar and starch contents under Pi depletion. Figure S5. PHR1 transcript or protein levels are not affected by high light exposure. Figure S6. Changes in subcellular Pi pool sizes upon short‐term high light. Figure S7. Monosaccharide levels after 20 min of high light. Figure S8. PSR marker genes are not induced in rosette leaves by sugar feeding. Figure S9. Anthocyanin biosynthetic and regulatory gene expression upon high light (HL). Figure S10. Anthocyanin production in response to sucrose is normal in the phr1‐1 single mutant. Figure S11. Total levels of lipid classes in WT (Col‐0) and srg3 mutants upon shift to high light (HL). Figure S12. Contents of glycosylglycerol lipid species in WT (Col‐0) and srg3 mutants upon shift to high light (HL) relative to fresh weight (FW). Figure S13. Contents of glycerophospholipid species in WT (Col‐0) and srg3 mutants upon shift to high light (HL) relative to fresh weight (FW). Figure S14. Levels of zeaxanthin and putative glycosylated SQDG determined in opposite ESI ion mode compared with Figure 7C. Figure S15. Levels of triacylglycerol (TAG) species in WT (Col‐0) and srg3 mutants upon shift to high light (HL) relative to fresh weight (FW). Figure S16. SRG3 gene expression upon light increase is not part of a heat response. [file TPJ-126-0-s003.pdf]

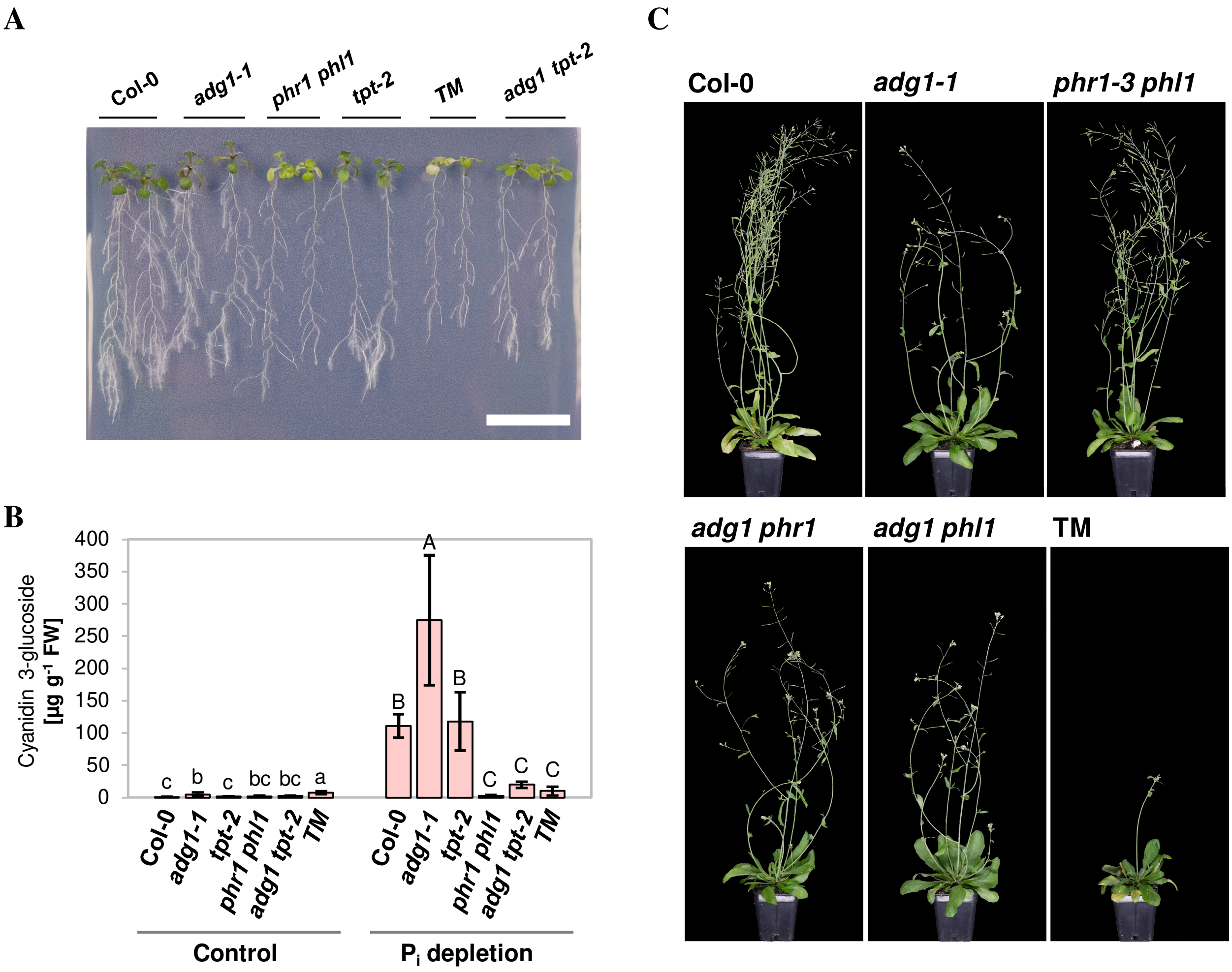

Supporting Information Fig. S1 Phenotypes of *adg1-1 phr1-3 phl1* (**TM**). **A**, Seedling phenotypes under P_i deficient growth conditions. Seedlings of WT (Col-0), *adg1-1*, *tpt-2*, *phr1-3 phl1*, *adg1-1 tpt-2* and TM genotype were grown for 7 days on rich medium (½ MS) including 0.5 % sucrose before transfer to media with 0.5 % sucrose and 0 mM (**P_i depletion**) KH_2PO_4 added. Growth was continued for 8 days. Bar, 2 cm. **B**, Anthocyanin (cyanidin 3-glucoside) contents of seedling shoots. Seedlings were grown and treated as described in A, but transfer to P_i-deferring media was conducted after 5 days of growth and treatment was sustained for 7 days. **Control**: 2.5 mM KH_2PO_4. Pigment levels are depicted relative to shoot fresh weights (**FW**). Bars represent means ± standard deviations; n = 5-6 pools of seedling shoots from 3 independent experiments. One-way ANOVA with Tukey HSD follow-up test and Bonferroni alpha correction for contrasts; $P < 0.05$. **C**, Inflorescences of WT (Col-0), *adg1-1*, *phr1-3 phl1*, *adg1 phr1*, *adg1 phl1*, and TM showing delay of the transition to flowering in the TM. Plants were grown under a 16-h light regiment at 23°C and a light intensity of 100±10 µmol m^{-2} s^{-1}. Pictures were taken of representative plants after 42 days of growth. Backgrounds were manually removed for better visualization.

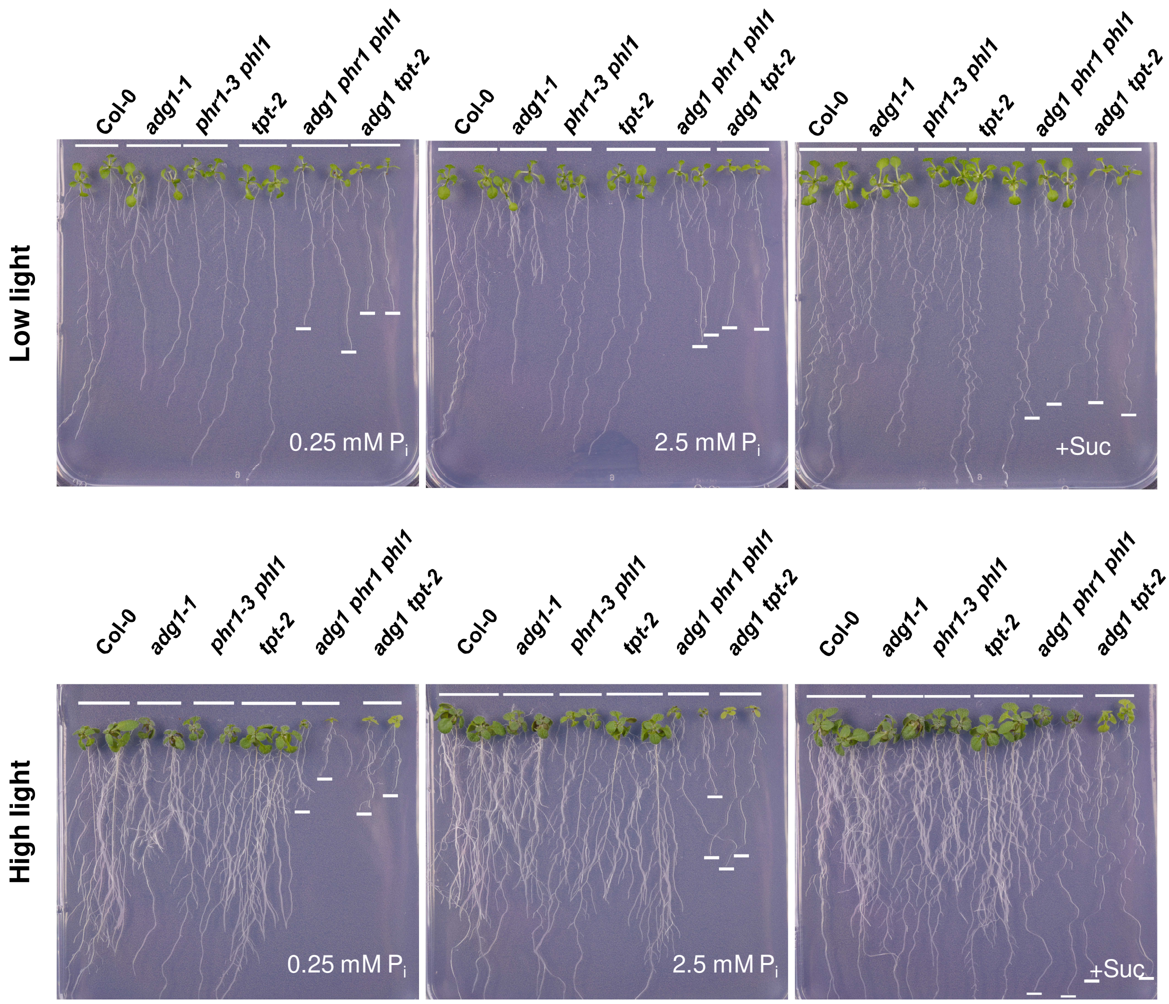

Supporting Information Fig. S2 Root growth of *adg1 phr1 phl1* and *adg1 tpt-2* responds to exogenously applied sucrose. Pictures show the seedling habitus of WT (Col-0), *adg1-1*, *phr1-3 phl1*, *tpt-2*, *adg1 phr1 phl1*, and *adg1 tpt-2*. Seedlings were grown as described in Figure 1D. White bars indicate apical ends of the primary roots of *adg1 phr1 phl1* and *adg1 tpt-2* lines. The experiment was performed 3 times with each 8 seedlings per condition. Representative pictures are shown.

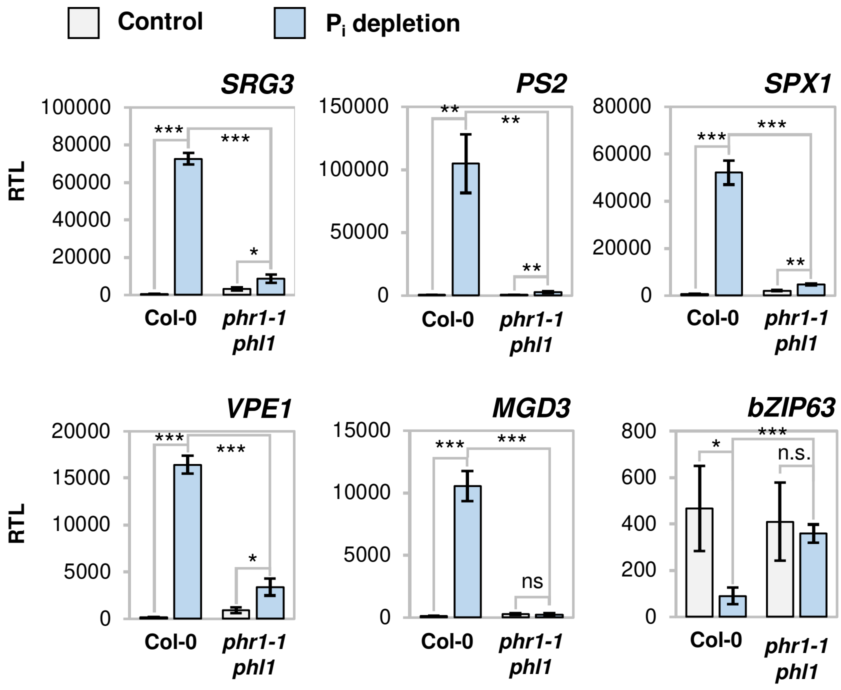

Supporting Information Fig. S3 Expression of selected P_i-starvation responsive genes depends on PHR1/PHL1 under P_i depletion. qRT-PCR analysis of *SRG3*, *PS2*, *SPX1*, *VPE1, MGD3*, and *bZIP63* transcript levels in seedlings of WT (Col-0) and *phr1-1 phl1* mutant genotype. Seedlings were grown on rich medium for 7 days before transfer to media with either 2.5 mM (Control, grey bars) or 0 mM (P_i depletion, blue bars) KH_2PO_4 added. Shoot material was harvested after additional 8 days of growth 9 h after onset of the 16-h photoperiod. Transcript levels were calculated relative to the transcript levels of *PP2A* as $1000{\cdot}2^{-\Delta CT}$. Bars show means ± standard deviations. $n = 3$ independent experiments. One-way ANOVA with Tukey HSD follow-up test and Bonferroni alpha correction for contrasts; ***$P < 0.001$, **$P < 0.01$, *$P < 0.05$, n.s., not significant.

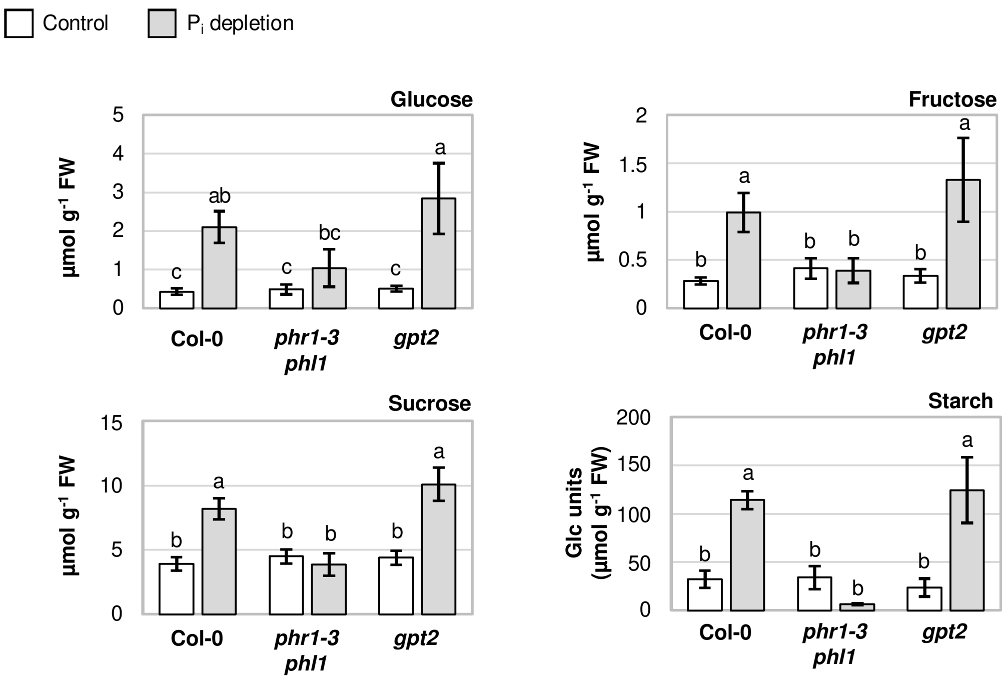

Supporting Information Fig. S4 Changes in sugar and starch contents under P_i depletion relative to fresh weight (**FW**). Seedlings of WT (Col-0), *phr1-3 phl1* and *gpt2* mutant genotype were grown for 10 days on rich medium before transfer to media containing either 2.5 mM (Control, white bars) or 0 mM (P_i depletion, grey bars) KH_2PO_4. Contents of glucose, fructose, sucrose and starch in seedling shoots were determined after additional 7 days of growth. Shoots were harvested 10.25 h after onset of the 16-h photoperiod. Bars represent means ± standard deviations; $n = 3$ independent experiments; 2-factor ANOVA with Tukey HSD post-hoc test; $P < 0.05$.

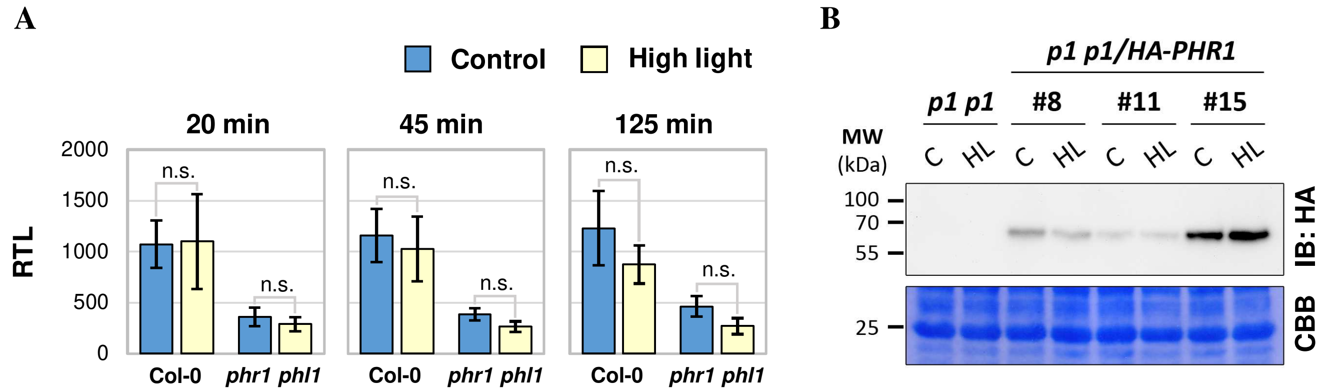

Supporting Information Fig. S5 *PHR1* transcript or protein levels are not affected by high-light exposure. **A**, qRT-PCR analysis of *PHR1* transcripts in rosette leaves from WT (Col-0) and *phr1-1 phl1* mutants. Plants were grown and treated as described in Figure 4. Transcripts were calculated relative to *PP2A* as $1000 \cdot 2^{-\Delta CT}$. Residual *PHR1* expression seen in *phr1-1 phl1* double mutants corresponds to missense transcript containing an early stop codon (Bustos et al., 2010). Bars represent means ± standard deviations; n = 3 independent experiments. Statistical analyses were performed using Student's t test with 2-tailed distribution; n.s., not significant. **B**, The *PHR1* genomic sequence was fused to a triple HA-tag sequence and expressed in the *phr1-1 phl1* (***p1 p1***) genomic background under control of a 1436 bp *PHR1* promotor fragment containing also 5' UTR and an intron upstream of ATG. Western blot analysis was performed on rosette leaves of 3 independent transgenic lines and the untransformed control line. Prior to harvest, plants were grown and treated with high light (**HL**) or control light intensity (**C**) as described for Figure 4. HA-PHR1 has a calculated molecular weight of 57 KDa.

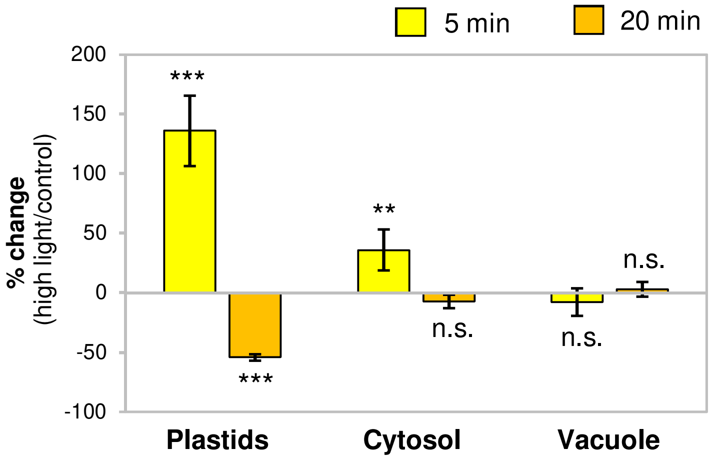

Supporting Information Fig. S6 Changes in subcellular P_i pool sizes upon short-term high light. Plants were grown and treated as described for Figure 4 and P_i levels were determined following non-aqueous fractionations. Percental changes in P_i levels of plastids, cytosolic/nuclear fraction, and vacuoles upon high-light treatments respective to controls are depicted. Data for 20 min are taken from the experiment shown in Figure 5A. Bars represent means ± standard deviations. n = 5 pools of rosettes from 3 independent trials (5 min) and 5 independent trials (20 min). Asterisks indicate significant differences between P_i levels upon high light and respective controls as per Student's t test with 2-tailed distribution at ***P < 0.001, **P < 0.01, n.s., not significant.

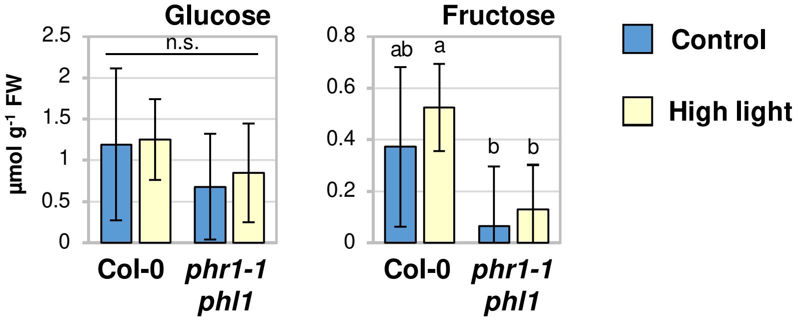

Supporting Information Fig. S7 Monosaccharide levels after 20 min of high light relative to fresh weight (**FW**). Plants were grown and treated as described in Figure 4. n = 8 plants from 4 independent experiments; bars show means ± standard deviations; 2-factor ANOVA with Tukey HSD post-hoc test; P < 0.05, n.s., not significant.

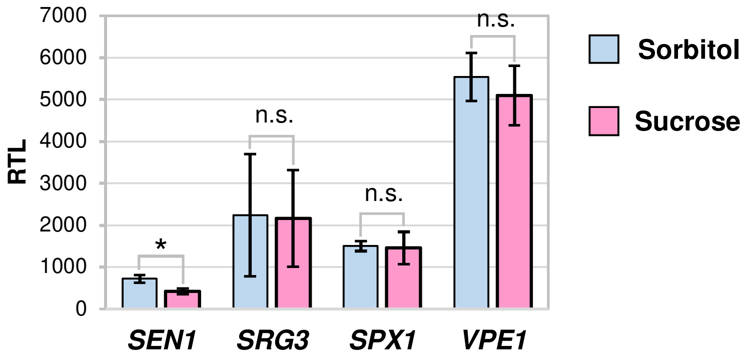

Supporting Information Fig. S8 PSR marker genes are not induced in rosette leaves by sugar feeding via the petioles. WT plants were grown as described in Figure 4. One hour into the photoperiod at 4 weeks age, rosette leaves were cut at the distal part of the petioles and placed into a buffered solution (10 mM MES, pH = 5.5; 1 mM $CaCl_2$) containing either 90 mM sucrose or 160.7 mM sorbitol (same osmotic strength), while the leaves were maintained under growth radiation of 70±5 µmol m^{-2} s^{-1} . After 30 min of exposure, leaves were harvested for qRT-PCR analysis. Transcript levels were calculated relative to *PP2A PP2A* as $1000 \cdot 2^{-\Delta CT}$. Bars represent means ± standard deviations; n = 3 independent experiments; statistical analyses were performed using Student's t test with 2-tailed distribution, unpaired with unequal variance. Significant differences are indicated as *P < 0.05, n.s., not significant.

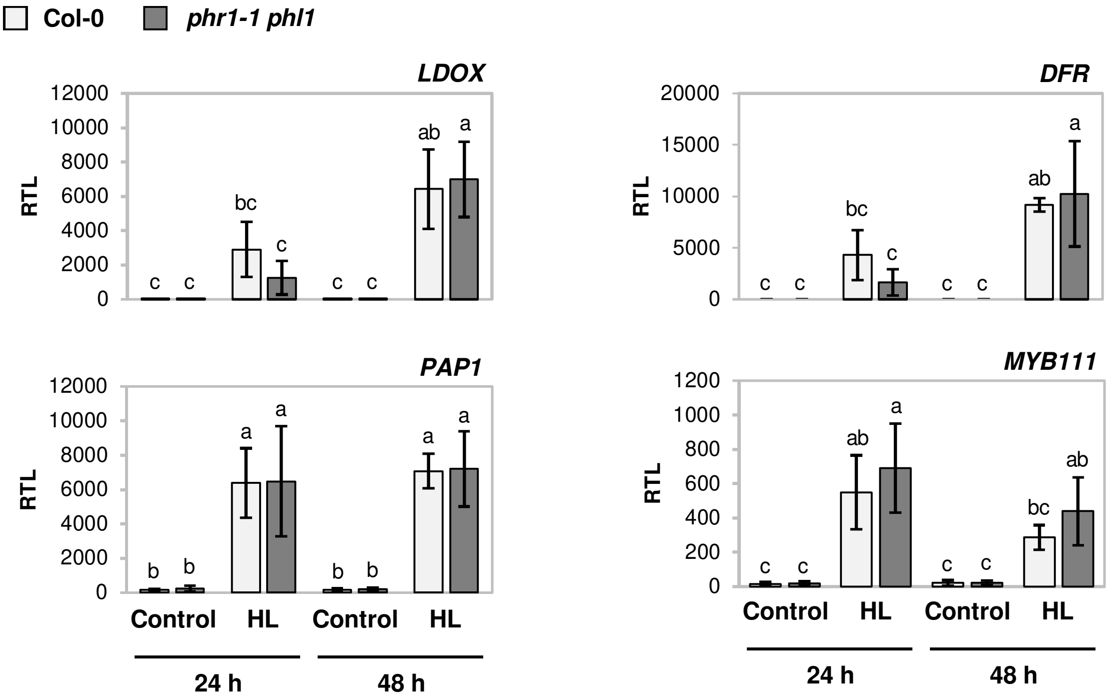

Supporting Information Fig. S9 Anthocyanin biosynthetic and regulatory gene expression upon high light (**HL**). WT (Col-0) and *phr1-1 phl1* mutants were grown for 39 days as described for Figure 4 before light intensity was shifted to 450±30 µmol m^{-2} s^{-1} (high light) at 4 h after onset of the photoperiod. Control plants were kept under growth light conditions (70±5 µmol m^{-2} s^{-1}). Material was harvested after 24 and 48 h. Transcript levels of *LDOX*, *DFR*, *PAP1*, and *MYB111* were calculated relative to *PP2A* as $1000 \cdot 2^{-\Delta CT}$. Bars show means ± standard deviations; $n = 3$ independent experiments; 2-factor ANOVA with Tukey HSD post-hoc test; $P < 0.05$.

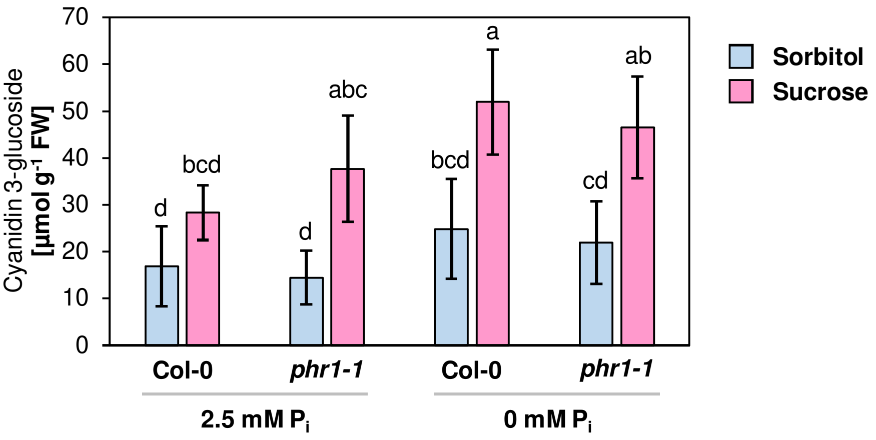

Supporting Information Fig. S10 Anthocyanin production in response to sucrose is normal in the *phr1-1* single mutant. Seedlings were grown on nutrient-rich media (no sucrose or sorbitol added) for 7 days under a 16-h light regime before transfer to media with either 0 or 2.5 mM KH_2PO_4 added and containing either 90 mM sucrose, or 160.7 mM sorbitol. Seedling shoots were harvested for anthocyanin determination after 67 h of growth on differing media. Anthocyanin (cyanidin 3-glucoside) contents are show relative to fresh weight (**FW**). $n = 4$ pools of seedling shoots from 4 independent experiments; 2-factor ANOVA with Tukey post-hoc test; $P < 0.05$.

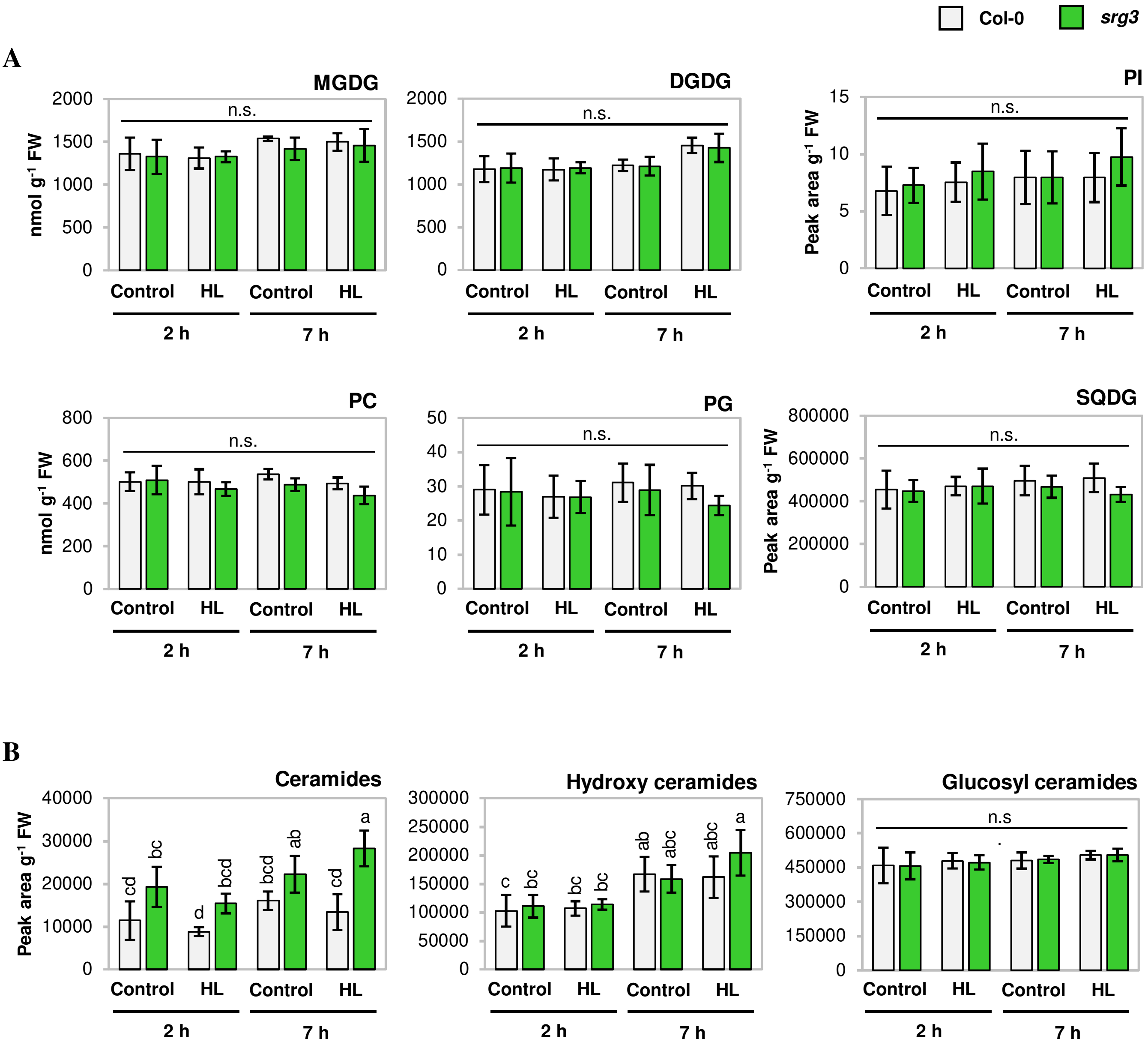

Supporting Information Fig. S11 Total levels of lipid classes in WT (Col-0) and *srg3* mutants upon shift to high light (**HL**). Plants were grown and treated as described for Figure 4. Rosette leaves (2-3 per sample) were harvested after 2 or 7 h of treatment. Contents were calculated relative to fresh weights (**FW**). Bars represent means ± standard deviations; n = 4 independent experiments; 2-factor ANOVA with Tukey HSD post-hoc test; $P < 0.05$; n.s., not significant. **A**, MGDG, Monogalactosyl diacylglycerol; DGDG, Digalactosyl diacylglycerol; PI, Phosphatidylinositol; PC, Phosphatidylcholine; PG, Phosphatidylglycerol; SQDG, Sulfoquinovosyl diacylglycerol. **B**, Levels of ceramides and of the ceramide derivatives hydroxy ceramides and glucosyl ceramides.

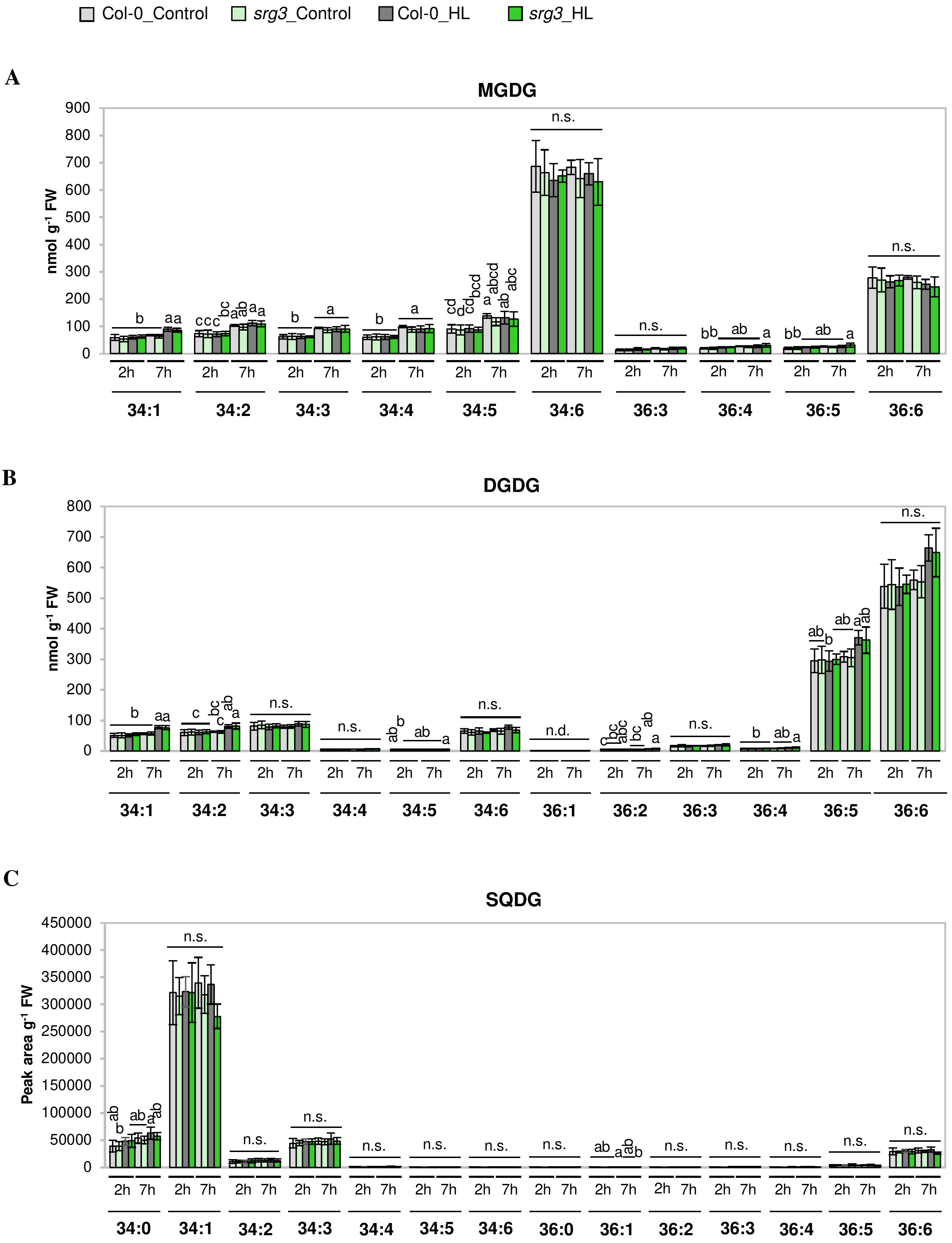

Supporting Information Fig. S12 Contents of glycosylglycerol lipid species in WT (Col-0) and *srg3* mutants upon shift to high light (**HL**) relative to fresh weight (**FW**). Plants were grown and treated as described for Figure S11. Bars represent means ± standard deviations; $n = 4$ independent experiments; 2-factor ANOVA with Tukey HSD post-hoc test; $P < 0.05$; n.s., not significant. n.d., not detected. **MGDG**, Monogalactosyl diacylglycerol; **DGDG**, Digalactosyl diacylglycerol; **SQDG**, Sulfoquinovosyl diacylglycerol.

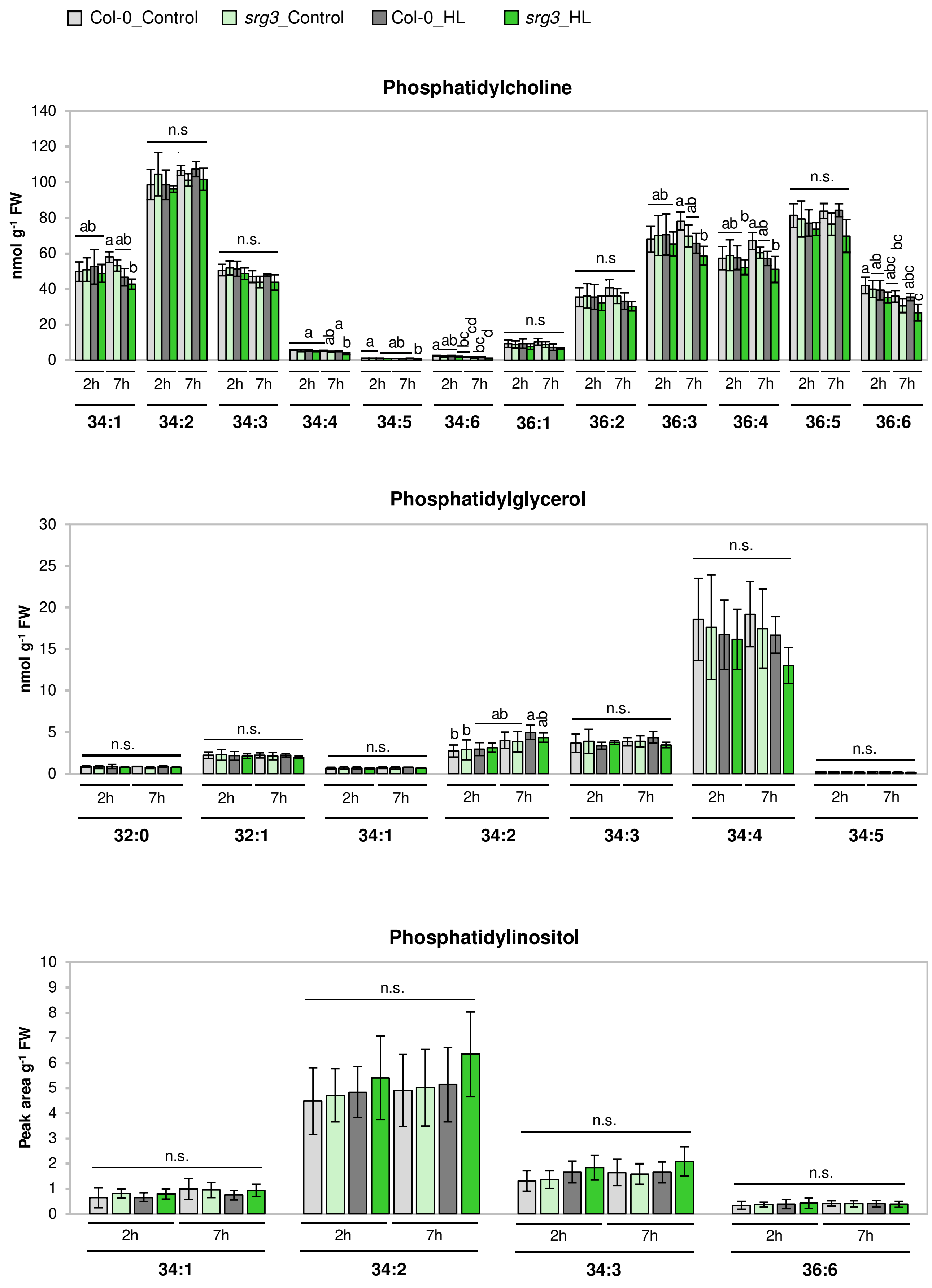

Supporting Information Fig. S13 Contents of glycerophospholipid species in WT (Col-0) and *srg3* mutants upon shift to high light (**HL**) relative to fresh weight (**FW**). Plants were grown and treated as described for Figure S11. Bars represent means ± standard deviations; *n* = 4 independent experiments; 2-factor ANOVA with Tukey HSD post-hoc test; $P < 0.05$; n.s., not significant.

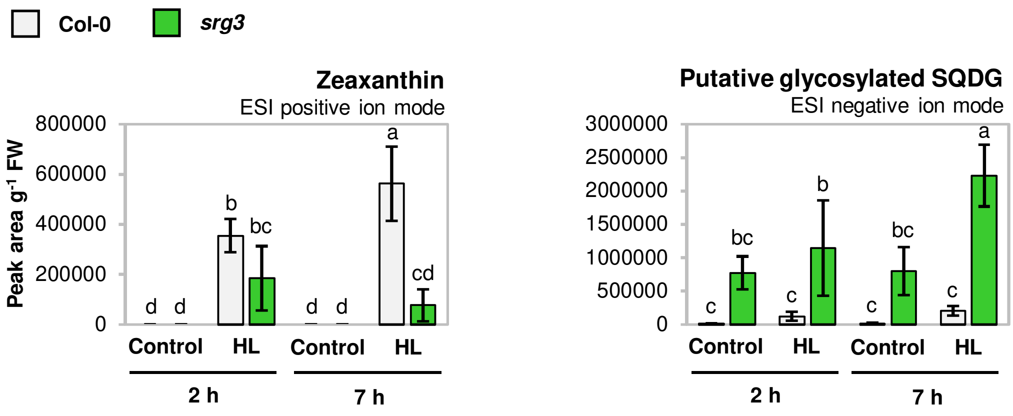

Supporting Information Fig. S14 Levels of zeaxanthin and putative glycosylated SQDG determined in opposite ESI ion mode compared to Fig. 7C and calculated relative to fresh weight (**FW**). Plants of WT (Col-0) and *srg3* mutant genotype were grown and treated as described for Figure S11. **HL**, High light. Bars represent means ± standard deviations; $n = 4$ independent experiments; 2-factor ANOVA with Tukey HSD post-hoc test; $P < 0.05$.

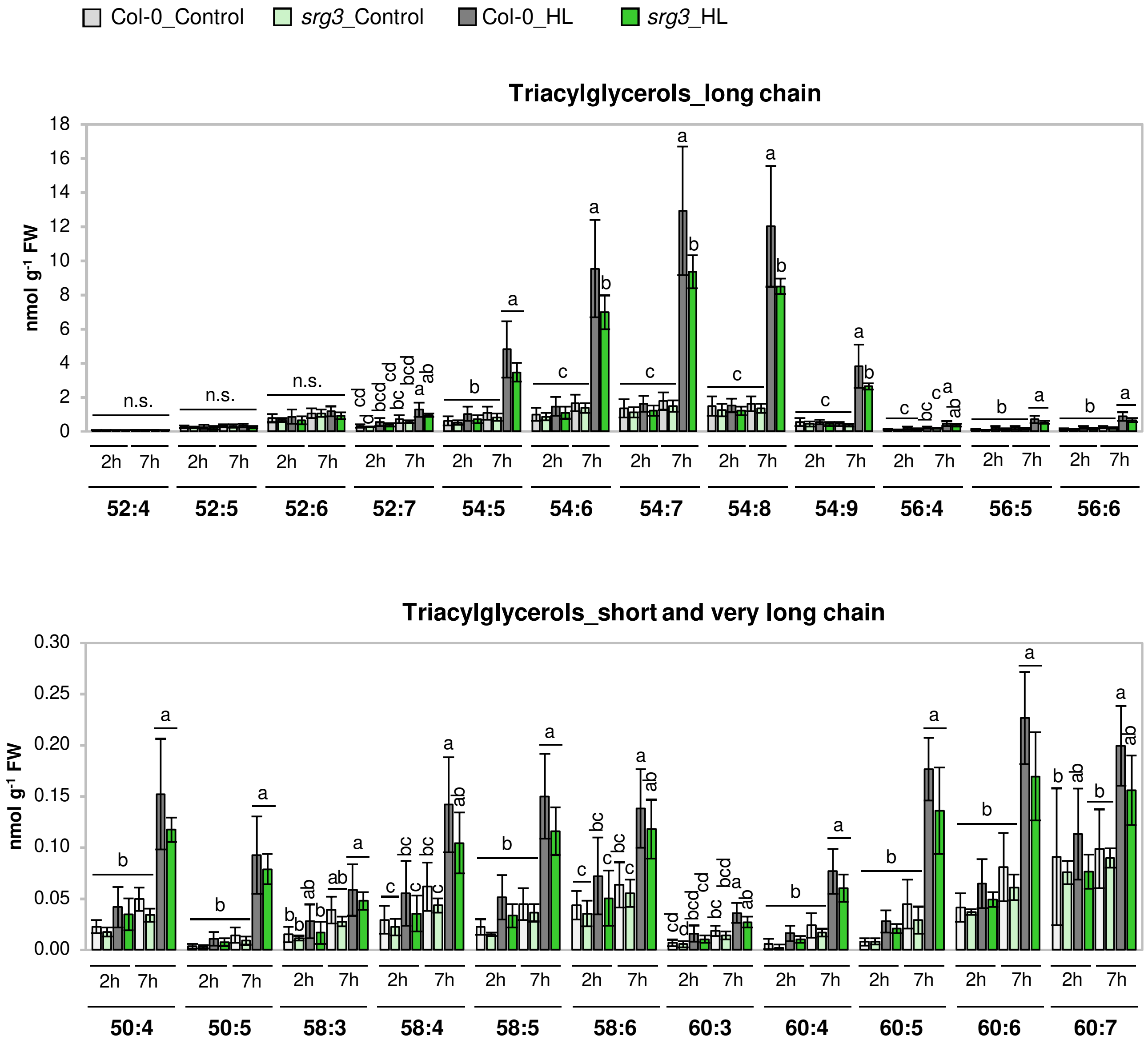

Supporting Information Fig. S15 Levels of triacylglycerol species in WT (Col-0) and *srg3* mutants upon shift to high light (**HL**) relative to fresh weight (**FW**). Plants were grown and treated as described for Figure S11. Bars represent means ± standard deviations; $n = 4$ independent experiments; 2-factor ANOVA with Tukey HSD post-hoc test; $P < 0.05$; n.s., not significant.

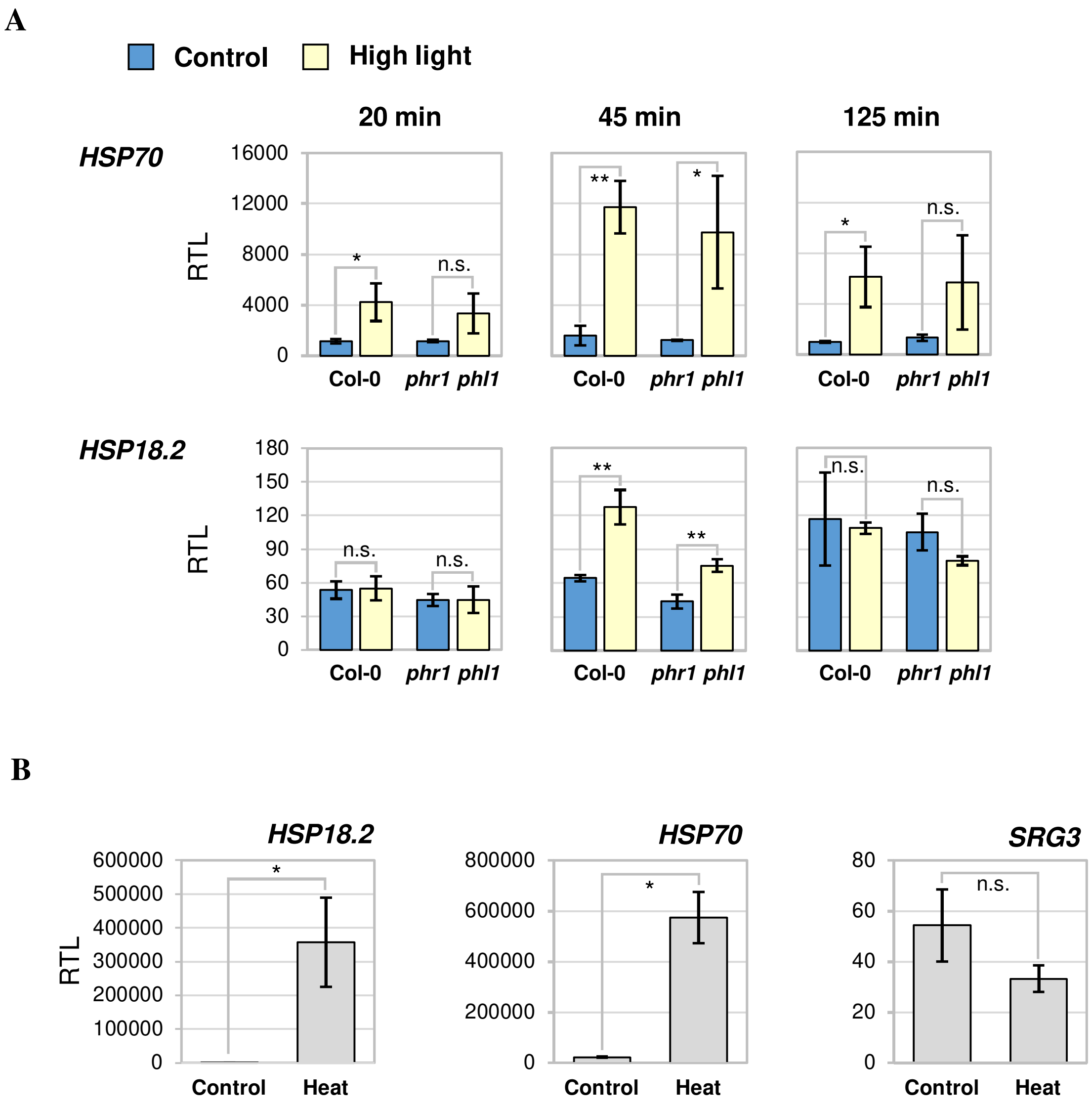

Supporting Information Fig. S16 *SRG3* gene expression upon light increase is not part of a heat response. Transcript levels were calculated relative to *PP2A* as $1000 \cdot 2^{-\Delta CT}$. Bars represent means ± standard deviations, $n = 3$ independent experiments. Statistical analyses were performed using Student's *t* test with 2-tailed distribution, unpaired with unequal variance. Significant differences are indicated as $^{**}P < 0.01$, $^{*}P < 0.05$, n.s., not significant. **A**, Levels of high-temperature responsive transcripts *HSP70* and *HSP18.2* in WT (Col-0) and *phr1-1 phl1* mutant plants upon high light treatment. Plants were grown and treated as described in Figure 4. Rosette leaves were sampled after 20, 45, and 125 min of treatment. **B**, *HSP18.2*, *HSP70,* and *SRG3* gene expression relative to *PP2A* in rosette leaves of WT plants upon heat stress. Plants were grown as described in Figure 4 and subjected to heat stress (38-40°C) for 30 min or kept under growth temperature (23°C). Stress was applied at 1 h into the potoperiod.
