# Supplementary material for: PHR1 mediates rapid high light responses and acclimation to high photosynthetic activity
Source: Plant J. 2026 May 5;126(3):e70901. doi: 10.1111/tpj.70901 (PMC13139890; doi:10.1111/tpj.70901)
Supplement: Supplementary file 3 — Table S4. List of primer sequences used in this study. [file TPJ-126-0-s004.pdf]

Supporting Information Table S4 List of primer sequences used in this study. Non-annealing regions are indicated by shading.

Primers	Sequence
**Genotyping primers**	
PHR1_F	5’ AAA CGA AGT GAT TGG CAT GAA 3’
PHR1_R	5’ AAG CGG TGT CAA CTT CCT TTC 3’
LBb1	5’ GCG TGG ACC GCT TGC TGC AAC T 3’
PHL1_F	5’ TCC CAC AAT CCA AAT TCA GAG 3’
PHL1_R	5’ CGG TTT ATA CCT TGC CGT TCT 3’
LB3	5’ GAAT TTCA TAA CCA ATC TCG ATA CAC 3’
NPTII_F	5’ TAT GAC TGG GCA CAA CAG ACA 3’
NPTII_R	5’ TTCA GCA ATA TCA CGG GTA GC 3’
TPT_F	5’ GGT GAT ATG GAA CTT CGA TTG G 3’
TPT_R	5’ GCG GTA TCT CCA CCT TCA GC 3’
*tpt-2*_F	5’ GTA ACT TAC GAG TAA ACT GGC TAC 3’
GPT2_F	5’ CGG TTT CTC AAG TCG GAC CA 3’
GPT2_R	5’ TGC TTC GCC TGC TCA ATG AT 3’
GK-LB	5’ ATA TTG ACC ATC ATA CTC ATT GC 3’
qSRG3_F	5’ CGG AGG AAC TGA GAT GTA CC 3’
SRG3_R	5’ ATC CTT ACA GAC CTC TCT TGC CC 3’
**qRT-PCR primers**	
qPP2A_F	5’ TAA CGT GGC CAA AAT GAT GC 3’
qPP2A_R	5’ GTT CTC CAC AAC CGC TTG GT 3’
qSPX1_F	5’ TCC AAG CAG AGT TAT CAG AGC AT 3’
qSPX1_R	5’ GGC GGC AAT GAA AAC ACA CT 3’
qMGD3_F	5’ ATC ACT AAG GCT GGT CCG GGT ACG 3’
qMGD3_R	5’ TGT CCA CAA CAT ACG GCA CGT TGC C 3’
qSRG3_F	5’ CGG AGG AAC TGA GAT GTA CC 3’
qSRG3_R	5’ CCG TAT GTC ATC AGA GAG AGC 3’
qVPE1_F	5’ CCT TAC TAA CGG GTT ACA TAT CG 3’
qVPE1_R	5’ GGC CTA GAC TCA GCT ATC TTC 3’
qPS2_F	5’ CTT GCC CTC CTA ACA TGT GC 3’

qPS2_R	5’ CTT GGA CAG TAA TCG CCA GC 3’
qbZIP63_F	5’ CGT TGA ATC GCA GTG CTT CC 3’
qbZIP63_R	5’ GGA GAC GGA AAC ACC ACA CG 3’
qLDOX_F	5’ TGA GCT AGC ACT CGG TGT GG 3’
qLDOX_R	5’ AAA GCT GCA AAC CCG GAA CC 3’
qDFR_F	5’ GGG TTT CAT CGG TTC ATG G 3’
qDFR_R	5’ AGT AGC GTC TTG GCG TTT GG 3’
qPAP1_F	5’ CTG GTC GGA CCG CAA ATG A 3’
qPAP1_R	5’ GGT GTT GTA GGA ATG GGC GT 3’
qPAP2_F	5’ GCC ACA ATA ACC CCC TAT TCC 3’
qPAP2_R	5’ CTC AAC CCT TTG GAC GAA CC 3’
qMYB111_F	5’ GAC CGA GAA GCA ATG GGA AG 3’
qMYB111_R	5’ CTT CCT CGG CTG TCC ATC TC 3’
qHSP70_F	5’ TGGGAATCAACTGGCTGAGG 3’
qHSP70_R	5’ TATCAGGCCCAGCTCCTTGG 3’
qHSP18.2_F	5’ AGC GGA GAG AGG AGC AAG GA 3’
qHSP18.2_R	5’ CGG AAC CAC AAC CGT AAG CA 3’
qSEN1_F	5’ TCC GAT GGA GGA AAG CAA CG 3’
qSEN1_R	5’ TCA CGC GCC ACT CTT ACT GG 3’
**Primers used for cloning**	
proGPT2_F	5’ ACC TGG ATC CGA ATG AAA ATG ACA AAC GAT ACA TTG 3’
proGPT2_R	5’ ATT GCC ATG GTG TGC TTT TTT ATG GCT AAT TGA TGA 3’
proSRG3_F	5’ ACC TGG ATC CCG CAG GTT GTC GAT ACA AAA G 3’
proSRG3_R	5’ ATT GCC ATG GAT TTC TAT TTT TAG AAA GAA AAA AGG GC 3’
proSPX1_F	5’ ACC TGA ATT CGT CGG TTC GGT TTG GTT CTG 3’
proSPX1_R	5’ ATT GCC ATG GAG CTC TTT TAT TTT CTG GGA AAC TTA A 3’
proSPX1_mut_F	5’ CAA GAA TAT TCC ATC GAA TCC AAC 3’
proSPX1_mut_R	5’ GTT GGA TTC GAT GGA ATA TTC TTG 3’
attB1-HA_FW	5’ AAA AAG CAG GCT TAA TGG GAT CCT ACC CAT ACG 3’
PHR1-HA_R	5’ CGA GCC TCT CCA GCA GAT CCA GCG TAA TCT GGA ACG TCG TAT G 3’
HA-PHR1_F	5’ GAT CTG CTG GAG AGG CTC GTC CAG TTC ATA G 3’
attB2-PHR1_R	5’ AGA AAG CTG GGT AGC CAG GTT TAC TAT TTA CTC ATA 3’
attB1_F	5’ GGG GAC AAG TTT GTA CAA AAA AGC AGG CT 3’

attB2_R	5’ GGG GAC CAC TTT GTA CAA GAA AGC TGG GT 3’
proPHR1_F	GGG GGC GCG CCG TAA GTT GCC ATT TTC ACT ATA AAT TTT G
proPHR1_R	CCC CTC GAG TGT TGT CCT GCA AGA GAG AAT CAT TAT CCA ACC
pNos_BlpI_F	AATGAGCTAAGCACATACGTCAG
NPTII_pNos_R	CAATCCCCATGGTAGATCCCCCTCGATCG
pNos_NPTII_F	GGGATCTACCATGGGGATTGAACAAGATGGATTG
NPTII_R	TGAACGATCTGCTTGACTCTAGGGGTCATCAGAAGAACTCGTCAAGAAGGC
AflII_adapter_F	CAATCTTAAGAAACTTTATTGCCAAATGTTTGAACGATCTGCTTGACTCTAG
